# Thymine DNA glycosylase mediates chromatin phase separation in a DNA methylation–dependent manner

**DOI:** 10.1016/j.jbc.2023.104907

**Published:** 2023-06-10

**Authors:** Lauren A. McGregor, Charles E. Deckard, Justin A. Smolen, Gabriela M. Porter, Jonathan T. Sczepanski

**Affiliations:** Department of Chemistry, Texas A&M University, College Station, Texas, USA

**Keywords:** thymine DNA glycosylase, liquid–liquid phase separation, DNA repair, biological condensate, DNA demethylation

## Abstract

Thymine DNA glycosylase (TDG) is an essential enzyme involved in numerous biological pathways, including DNA repair, DNA demethylation, and transcriptional activation. Despite these important functions, the mechanisms surrounding the actions and regulation of TDG are poorly understood. In this study, we demonstrate that TDG induces phase separation of DNA and nucleosome arrays under physiologically relevant conditions *in vitro* and show that the resulting chromatin droplets exhibited behaviors typical of phase-separated liquids, supporting a liquid–liquid phase separation model. We also provide evidence that TDG has the capacity to form phase-separated condensates in the cell nucleus. The ability of TDG to induce chromatin phase separation is dependent on its intrinsically disordered N- and C-terminal domains, which in isolation, promote the formation of chromatin-containing droplets having distinct physical properties, consistent with their unique mechanistic roles in the phase separation process. Interestingly, DNA methylation alters the phase behavior of the disordered domains of TDG and compromises formation of chromatin condensates by full-length TDG, indicating that DNA methylation regulates the assembly and coalescence of TDG-mediated condensates. Overall, our results shed new light on the formation and physical nature of TDG-mediated chromatin condensates, which have broad implications for the mechanism and regulation of TDG and its associated genomic processes.

Biological phase separation is a widely occurring biomolecular process that underlies the formation of membraneless organelles in cells (1, 2, 3). These protein-rich compartments, referred to as biological condensates, are often characterized as having liquid-like properties and are proposed to form through the physical process of liquid–liquid phase separation (LLPS). This phenomenon is increasingly recognized to play important roles in a wide range of biological processes, including chromatin organization (4), signal transduction (5), transcription (6), and DNA repair (7). Although the interactions driving the formation of these condensates, as well as the physical properties underlying their functions, remain poorly understood, the involvement of proteins with intrinsically disordered regions (IDRs) has emerged as a common theme (8). IDRs are defined as a stretch of amino acids with low sequence complexity and undefined secondary structures. IDRs also tend to have biased amino acid compositions, particularly those with polar, charged, and aromatic residues (9). Many studies have revealed that low-affinity multivalent interactions among these amino acids within IDRs are an essential driving force of LLPS and the assembly of biological condensates (1, 10, 11).

Thymine DNA glycosylase (TDG) has been shown to recognize and excise mismatched pyrimidine bases from G•T and G•U pairs in order to initiate base excision repair (BER) at these sites (12, 13). Moreover, as the only known enzyme capable of removing the DNA demethylation intermediates, 5-formalcytosine and 5-carboxylcytosine, from DNA in mammals, TDG plays an essential role in epigenetic regulation (14, 15). In addition, TDG has been shown to potentiate transcription by coordinating the recruitment of various transcription factors and activating histone modifiers to target genes, resulting in local changes to the chromatin environment at both the epigenetic and structural levels (16, 17, 18, 19, 20). Recently, TDG inhibition was identified as a viable clinical strategy in melanoma (21). Given these important functions, it is critical that we establish the mechanisms surrounding the actions and regulation of TDG.

The majority of studies on TDG have focused on actions of its folded catalytic domain and glycosylase activity. However, the role of N- and C-terminal domains (CTDs) of TDG remains poorly understood and unexplored, representing a major gap in our understanding of this essential enzyme. Previous NMR studies have shown that N- and CTDs of TDG are intrinsically disordered (22, 23), which is also predicted based on their amino acid sequence (Fig. 1*A*). An intriguing hypothesis is that N- and C-terminal IDRs of TDG facilitate LLPS. The terminal IDRs of TDG account for more than half its mass, have low sequence complexity, and contains an abundance of charged and polar residues (∼60% total; Fig. S1), all sequence characteristics that are known to promote biomolecular phase separation, and specifically LLPS, especially in the presence of nucleic acids (1, 9, 24, 25). Indeed, we recently reported that TDG IDRs mediate the oligomerization of chromatin fibers into insoluble condensates (26). The ability of TDG to interact with many different proteins *via* its IDRs is also consistent with a phase-separation mechanism.

**Figure 1:**
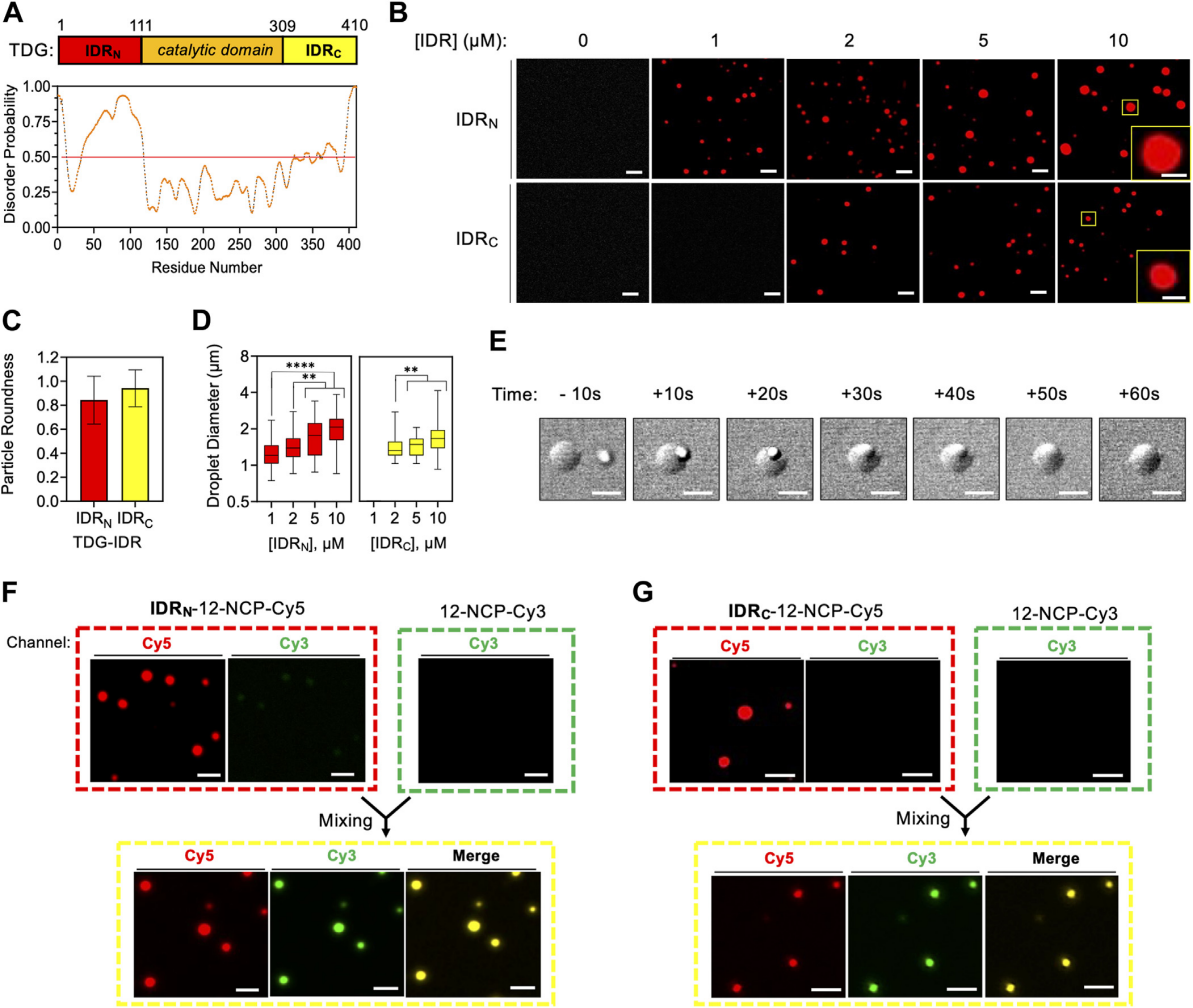
**TDG’s IDRs induce phase separation of chromatin.***A*, diagram of the structural domains of TDG and their predicted disorder probability (PrDOS) (68). *B*, representative confocal fluorescent microscopy images of 12-NCP-Cy5 chromatin (25 nM) in the presence of the indicated [IDR]. Droplets were formed in the presence of LLPS buffer (10 mM Hepes [pH 7.2], 100 mM KCl, and 1 mM MgCl_2_) containing 5% PEG. *C* and *D*, circularity and diameter of individual chromatin droplets formed by TDG’s IDRs. Data are mean ± SD (n > 600 droplets). ∗∗*p* < 0.01; ∗∗∗∗*p* < 0.0001. *E*, time-lapse images of an IDR_N_-chromatin droplet fusion event. Droplets were formed by combining 25 nM 12-NCP-Cy5 with 5 μM IDR_N_. *F* and *G*, representative confocal fluorescent microscopy images demonstrating that 12-NCP-Cy3 chromatin (50 nM) penetrates into preformed IDR_N_-12-NCP-Cy5 (*F*) and IDR_C_-12-NCP-Cy5 (*G*) droplets generated by mixing 5 μM of the IDR with 12.5 nM chromatin. Scale bars for *zoom insets* in *B* represents 2 μm. All other scale bars represent 5 μm. IDR, intrinsically disordered region; LLPS, liquid–liquid phase separation; TDG, thymine DNA glycosylase.

These observations, along with the potential implication for an LLPS model for TDG, motivated us to examine the phase behavior of TDG and the potential role of its IDRs. Herein, we show that TDG induces phase separation of DNA and nucleosome arrays under physiologically relevant conditions *in vitro* and provide evidence that the resulting biomolecular condensates have liquid-like properties, supporting an LLPS model for TDG. Evidence also suggests that TDG has the capacity to form phase-separated condensates in the cell nucleus. The ability of TDG to assemble chromatin condensates *in vitro* is regulated by its N- and C-terminal IDRs, which in isolation, produce chromatin-containing droplets with distinct physical properties, consistent with their unique mechanistic roles in this process. Finally, we demonstrate that TDG–chromatin condensates are sensitive to the methylation status of the DNA, supporting a role for 5-methylcystosine (5mC) in regulating the distribution of TDG–chromatin condensates throughout the nucleus. Overall, by demonstrating the ability of TDG to promote phase separation of chromatin, this study provides a new perspective on the mechanisms and regulation of TDG-mediated genomic processes.

## Results

### IDRs of TDGs induce phase separation of chromatin *in vitro*

Given the involvement of protein IDRs in LLPS, along with our prior observation that the isolated N-terminal domain of TDGs can induce chromatin condensation, we first examined the phase behavior of chromatin in the presence of TDG’s isolated N-terminal IDR (IDR_N_; residues 1–110) and C-terminal IDR (IDR_C_; residues 309–410) (Fig. 1*A*). For these experiments, we employed *in vitro* reconstituted nucleosome arrays consisting of 12 repeats of Widom’s 601 nucleosome positioning sequence, assembled using our previously described methods (Fig. S2) (26, 27). For visualization purposes, the arrays were reconstituted with either Cy3- or Cy5-labeled histone octamers, yielding chromatin labeled with the corresponding dye (12-NCP-Cy3 and 12-NCP-Cy5, respectively). Using confocal fluorescence microscopy, we found that both TDG’s IDRs induced the formation of micron-size droplets when mixed with a substoichiometric amount of 12-NCP-Cy5 chromatin under physiological salt conditions (Figs. 1*B* and S3, *A*–*D*). The chromatin droplets formed by TDG’s IDRs exhibited typical behaviors of phase-separated liquids, including a spherical shape (Fig. 1*C*) and the ability to rapidly (∼1 min) fuse with each other (Fig. 1*E*). Furthermore, droplet size and IDR concentration were positively correlated, with the IDR_N_ producing larger droplets than the IDR_C_ at the highest concentrations tested (Fig. 1*D*). Droplet formation was not observed with either 12-NCP-Cy5 or TDG’s IDRs alone under identical conditions (Figs. 1*B* and S3*E*), indicating that this process requires both components under the conditions tested herein.

If phase-separated condensates have liquid-like properties, molecular exchange often occurs between the dense and light phases (12). To test this, we mixed preformed IDR-chromatin droplets with dilute propidium iodide (PI), a DNA-intercalating dye. During the time required for sample mixing and imaging (<2 min), a strong PI signal could be detected within the droplets that colocalized with 12-NCP-Cy5 (Fig. S4). Thus, small molecules can freely diffuse into chromatin condensates formed by TDG’s IDRs. However, for biological condensates to serve a functional purpose inside the cell, it is essential that biomacromolecules (*e.g.*, DNA and proteins) are similarly able to diffuse in (and out) of the condensed phase. With this in mind, we examined whether full-sized chromatin fibers (molecular weight: >2.5 × 10^6^ Da) could diffuse into the droplets. Using 12-NCP-Cy5 arrays, we generated condensates with either the IDR_N_ or the IDR_C_ and then, after visually confirming the presence of droplets, added an equivalent of an orthogonally labeled nucleosome array (12-NCP-Cy3). As with PI, the differentially labeled chromatin fibers colocalized within the droplets shortly after mixing (<2 min), indicating that 12-NCP-Cy3 rapidly diffused into and accumulated within the preformed 12-NCP-Cy5 condensates (Fig. 1, *F* and *G*). The results of these mixing experiments, combined with the ability of droplets to undergo fusion, suggests that chromatin condensates mediated by TDG’s IDRs can grow in size by either merging with other droplets or by accumulating more chromatin molecules.

### TDG’s IDRs generate phase-separated condensates with distinct material properties

**Figure 2:**
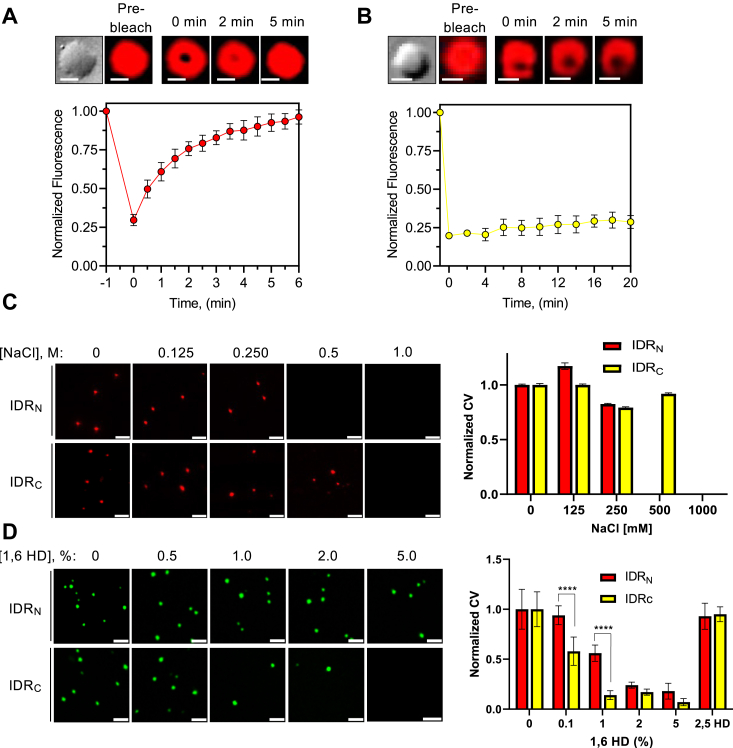
**Unique phase behavior of TDG’s IDRs.***A* and *B*, confocal fluorescent microscopy images and FRAP curve of 12-NCP-Cy3 chromatin condensates formed by IDR_N_ (*A*) and IDR_C_ (*B*). Droplets were formed by combining 25 nM 12-NCP-Cy3 with 5 μM IDR_N_ or 10 μM IDR_C_. Data are mean ± SD (n = 3). Scale bars represent 5 μm. *C*, confocal fluorescence microscopy images and normalized CV analysis of 12-NCP-Cy5 chromatin (25 nM) condensates formed by TDG’s IDRs (5 μM) following the addition of NaCl. Data are mean ± SD (n = 10 images). *D*, confocal fluorescence microscopy images and normalized CV analysis of 12-NCP-Cy3 chromatin (25 nM) condensates formed by TDG’s IDRs (5 μM) following the addition of 1,6-HD. Data are mean ± SD (n = 10 images). ∗∗∗∗*p* < 0.0001. All scale bars represent 5 μm. Buffer conditions are the same as described for Figure 1, except where indicated. 1,6-HD, 1,6-hexanediol; FRAP, fluorescence recovery after photobleaching; IDR, intrinsically disordered region; TDG, thymine DNA glycosylase.

To further probe the material properties of chromatin condensates formed by TDG’s IDRs, we employed fluorescence recovery after photobleaching (FRAP) as a tool to study internal droplet dynamics (28). Chromatin droplets (12-NCP-Cy3) induced by IDR_N_ showed almost full recovery of partially bleached fluorescence after 300 s (Fig. 2*A*), suggestive of a liquid-like state. In contrast, chromatin droplets (12-NCP-Cy3) induced by IDR_C_ failed to recover (Fig. 2*B*), indicating that their internal dynamics are very slow relative to droplets formed with IDR_N._ This behavior is more consistent with a bridged polymer scaffold rather than a liquid (29). We note that, similar to a liquid, the more rigid structures formed by polymer bridging still permit rapid molecular exchange with the light phase, as observed previously (Fig. 1, *F* and *G*). The different recovery kinetics between droplets formed by IDR_N_ and IDR_C_ is likely reflective of their distinct nature and strength of interaction, as the two IDRs have different amino acid sequences (Fig. S1) (1, 30). For example, while both IDRs have a high fraction of charged residues, their net charge per residue (31) varies greatly, with IDR_N_ being overall slightly cationic and IDR_C_ being overall anionic. Such differences are expected to give rise to distinct interaction modes and material properties within condensates comprising negatively charged chromatin.

We next sought to probe the type of interactions underlying the distinct droplet dynamics observed previously. We first titrated salt (NaCl), which revealed that high-salt concentrations inhibited phase separation of 12-NCP-Cy5 chromatin by both IDRs. Chromatin droplets formed by IDR_N_ were more sensitive to high salt than those formed by IDR_C_ (Fig. 2*C*), suggesting that ionic interaction plays a greater role for IDR_N_. We also examined droplet formation in the presence of 1,6-hexanediol (1,6-HD), an aliphatic alcohol that disassembles phase-separated condensates by disrupting hydrophobic interactions (32). Droplets formed by IDR_N_ were generally more resistant to 1,6-HD treatment than those formed by IDR_C_. In fact, chromatin droplets formed by IDR_C_ were almost completely disassembled in the presence of >1% 1,6-HD (Fig. 2*D*). Little effect was observed by the similar aliphatic alcohol 2,5-hexanediol (2,5-HD), which has minimal impact on the phase behavior of disordered proteins (33, 34). Together, these results suggest that, while both electrostatic and hydrophobic interactions contribute to the formation of chromatin droplets by TDG’s IDRs, IDR_N_ is more reliant on electrostatic interactions, whereas hydrophobic interactions play a greater role in phase separation for IDR_C_. The larger contribution of hydrophobic interactions to the stability of condensates formed by IDR_C_ possibly explains their reduced internal dynamics (Fig. 2*B*) (1). We note that these studies also demonstrate that chromatin condensates formed by both TDG’s IDRs are reversible.

### Full-length TDG induces phase separation of genomic DNA *in vitro*

**Figure 3:**
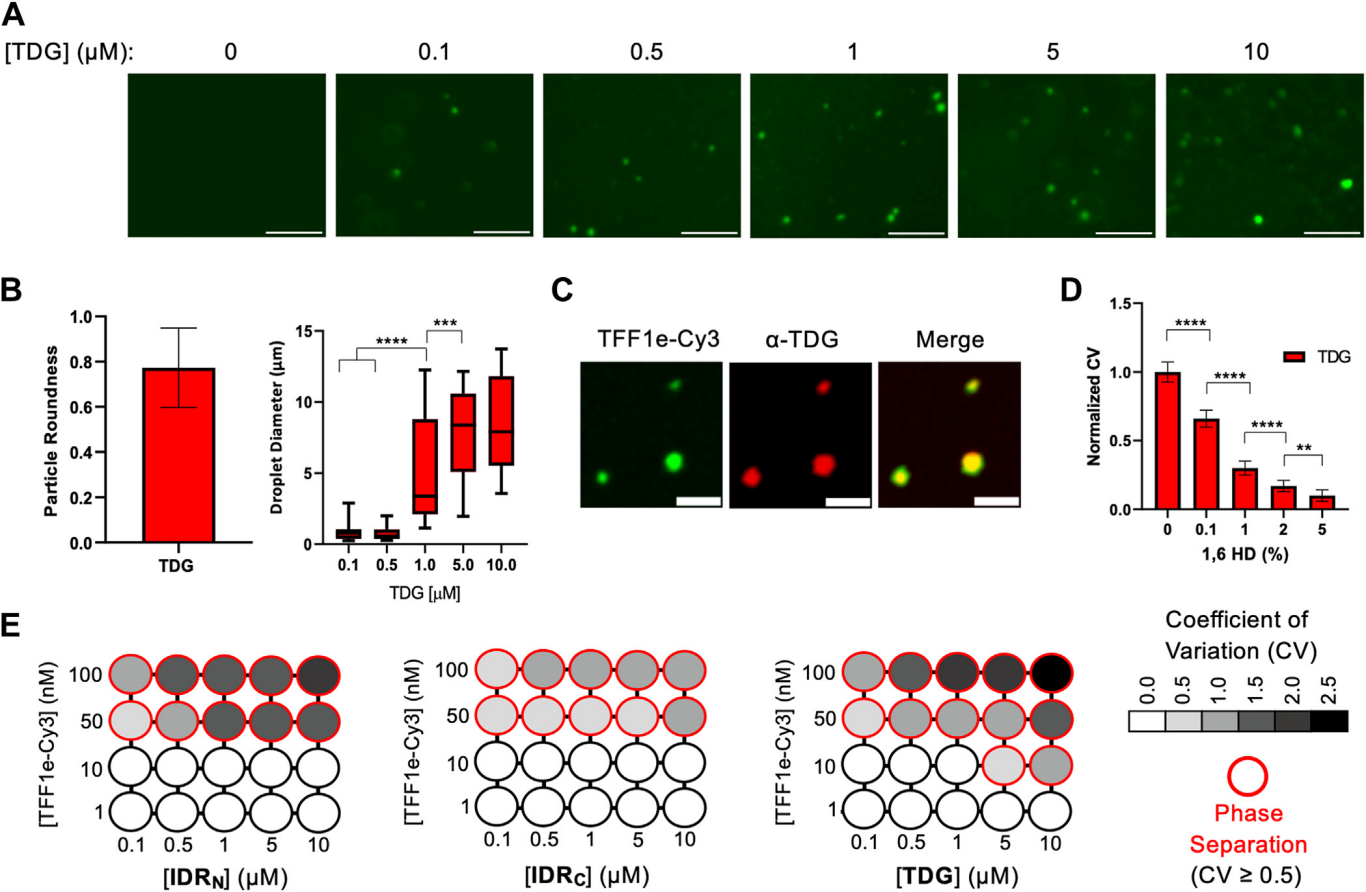
**Full-length TDG induces phase separation of TFF1-derived chromatin.***A*, representative wide-field fluorescent microscopy images of TFF1e-Cy3 chromatin (100 nM) in the presence of the TDG. Scale bars represent 5 μm. *B*, Circularity and diameter of individual chromatin droplets formed by TDG. Data are mean ± SD (n > 600 droplets). *C*, representative wide-field fluorescent microscopy images demonstrating that Cy5-labled anti-TDG antibody (α-TDG_360–410_) penetrates into preformed TFF1e-Cy3-TDG droplets generated by mixing 1 μM TDG with 25 nM chromatin. Scale bars represent 5 μm. *D*, normalized CV analysis of TFF1e-Cy3 chromatin (25 nM) condensates formed by TDG (5 μM) following the addition of 1,6-HD. Data are mean ± SD (n = 10 images). ∗∗*p* < 0.01; ∗∗∗∗*p* < 0.0001. *E*, phase diagrams of TFF1e-Cy3 chromatin under varying conditions. *Red circles* indicate phase separation. The *grayscale* indicates CV calculated from representative images (n = 10; Fig. S8). 1,6-HD, 1,6-hexanediol; TDG, thymine DNA glycosylase.

We next shifted our attention to the full-length TDG protein. For these studies, we examined the ability of TDG to induce phase separation of chromatin comprising a native DNA sequence, namely the *TFF1* gene enhancer (*TFF1e*). The *TFF1e* is an ideal model for these studies because it is bound by TDG *in vivo* and undergoes TDG-dependent promoter–enhancer looping in estrogen-positive tissues upon treatment with 17β-estradiol (E2), a process that recent studies suggest involves phase separation (20, 34). We also sought to demonstrate that the TDG-mediated chromatin condensates were not artifacts of the 601 DNA sequence or corresponding 12-mer nucleosome arrays. To examine the interaction of TDG with *TFF1e* in isolation, we reconstituted Cy3-labeled human histones with a ∼2500 bp region of *TFF1e via* salt dialysis to yield the corresponding chromatin (TFF1e-Cy3) (Fig. S5). Mixing of TDG with TFF1e-Cy3 chromatin under physiological conditions resulted in the formation of quantitatively round droplets analogous to those generated by TDG’s individual IDRs (Fig. 3, *A* and *B*). We confirmed that TDG localized within TFF1e-Cy3 droplets *via in situ* immunostaining with Cy5-conjugated antibodies specific for TDG (α-TDG_360–410_) (Figs. 3*C* and S6). At TDG concentrations ≥5 μM, we occasionally observed a very small number of TDG droplets by *in situ* immunostaining in the absence of chromatin, which failed to meet our definition of phase separation (CV >0.5) (Fig. S6). Thus, while we cannot unequivocally rule out that TDG phase separates on its own, TDG was unable to undergo phase separation in the absence of chromatin under the conditions tested herein. Chromatin droplets formed by TDG were also reversible by 1,6-HD treatment (Figs. 3*D* and S7), indicating that, like the isolated IDRs, hydrophobic interactions are a major driving force for assembly of chromatin condensates by the full-length protein.

To garner further insights into the nature of the TDG–chromatin condensates, we generated a phase diagram by systematically varying the concentration of TFF1e-Cy3 chromatin and TDG (Figs. 3*E* and S8). We quantified phase separation by measuring the heterogeneity (CV) of fluorescent intensities across multiple images (n = 10). The higher the CV, the greater heterogeneity of TFF1e-Cy3 signal (*i.e.*, phase separation). Compared with the isolated IDRs, full-length TDG formed condensates at much lower chromatin concentrations, possibly reflecting its enhanced affinity for DNA imparted by the catalytic domain. TFF1e-Cy3 chromatin did not phase separate by itself under any condition tested (Fig. S8). At chromatin concentrations ≥50 nM, robust droplet formation occurred even at the lowest TDG concentration tested (100 nM) (Fig. 3*E*). The estimated concentration of TDG in human cell nuclei is ∼150 nM (35), indicating that physiologically relevant TDG concentrations are sufficient to induce assembly of phase-separated chromatin droplets. Moreover, the concentration regime in which we observe TDG-mediated chromatin phase separation (0.1–10 μM), as well as our protein-to-chromatin ratios (1:1–100:1), is similar to and in many cases lower than what has been reported for many other transcriptional regulators known to induce LLPS of chromatin *in vivo* (4, 36, 37, 38, 39).

### IDR_N_ and IDR_C_ have opposing roles in the process of chromatin phase separation

**Figure 4:**
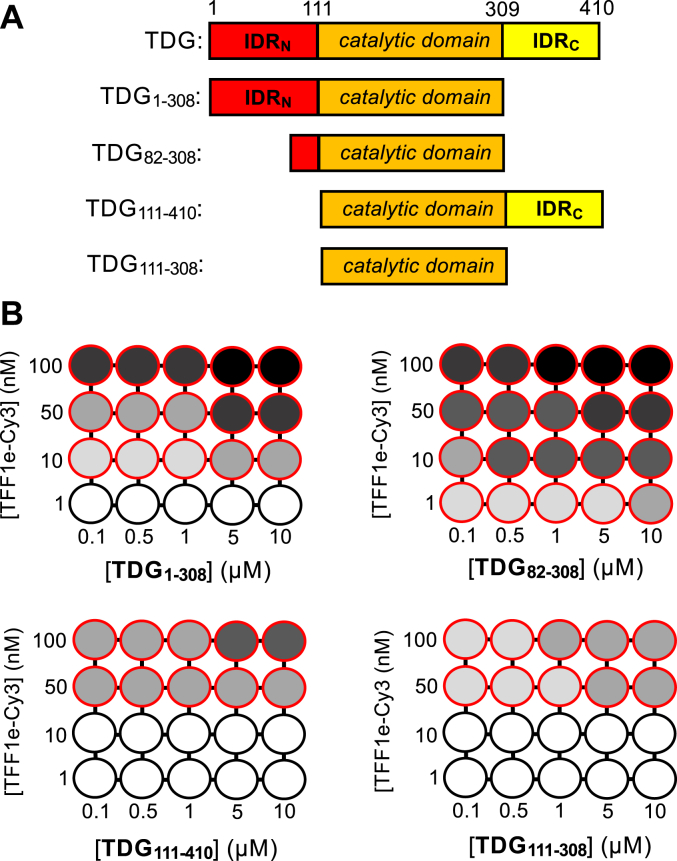
**TDG-mediated chromatin phase separation is regulated by its IDRs.***A*, TDG domains and truncated variants discussed in this work. *B*, phase diagrams of TFF1e-Cy5 chromatin and TDG truncations under varying conditions. *Red circles* indicate phase separation. The *grayscale* indicates CV as described for Figure 3*E* (Fig. S9). IDR, intrinsically disordered region; TDG, thymine DNA glycosylase.

We next sought to dissect the contribution of TDG individual domains toward inducing chromatin condensation using a series of truncated proteins (Fig. 4). Given the distinct phase behaviors of TDG’s isolated IDRs, we expected that these domains would contribute differently to the process. Indeed, we previously showed that TDG’s IDRs have contrasting roles in mediating the oligomerization of chromatin fibers into insoluble aggregates, which our current data suggest are actually phase-separated droplets (26). In our model, the polycationic IDR_N_, and in particular residues 82 to 110, bind DNA and/or protein surfaces between chromatin fibers through nonspecific interactions to facilitate condensation (*i.e.*, phase separation). In contrast, the IDR_C_ antagonizes this process by weakening interfiber interactions mediated by IDR_N_, potentially through direct contacts between the two disordered domains. Phase diagrams generated using various TDG truncations and TFF1e-Cy3 chromatin mostly corroborated this model (Figs. 4 and S9), although mechanisms that are independent of TDG-mediated interfiber contacts could also be involved. TDG variants lacking IDR_c_ but containing all (TDG_1–308_) or the most basic region of IDR_N_ (TDG_82–308_) had improved phase separation ability relative to the full-length protein, consistent with the antagonizing effects of IDR_C_ observed previously. Similar to IDR_C_, the first 50 residues of IDR_N_ are also known to destabilize DNA binding by TDG (23), which may explain why TDG_82–308_ has the greatest potential to induce chromatin phase separation of all truncations tested. Interestingly, the TDG variant lacking both IDRs (*i.e.*, the catalytic domain alone; TDG_111–308_) was still able to induce phase separation of chromatin, although at a greatly decreased level compared with variants containing IDR_N_. Previous studies have shown that the catalytic domain interacts weakly with DNA and itself (*i.e.*, dimerization), providing a potential driving force for phase separation (40, 41). The increased chromatin phase separation ability imparted by IDR_N_ (TDG_1–308_ and TDG_82–308_) is consistent with its ability to enhance nonspecific DNA binding and facilitate intermolecular interactions, both of which are expected to further promote chromatin condensation (23, 40, 41). In contrast, the phase separation ability of TDG_111-410_, which contains the catalytic domain and IDR_C_, but not IDR_N_, was similar to the catalytic domain alone (TDG_111–308_). Thus, despite the ability of IDR_C_ to induce chromatin phase separation in isolation (Fig. 1*B*), it appears to contribute minimally to this process in the context of the full-length protein. Instead, its primary role may be to antagonize interactions mediated by IDR_N_ and the catalytic domain in order to fine-tune conditions needed to induce phase separation of chromatin. However, we cannot rule out other roles for IDR_C_, such as controlling the material properties of TDG–chromatin condensates. Overall, these data show that, while the catalytic domain is sufficient for TDG-induced phase separation of chromatin *in vitro*, the combined activities of IDR_N_ and IDR_C_ allow for fine-tuning of this process by either promoting or impeding phase separation, respectively (Fig. S10). In the future, it will be important to determine the exact nature of the interactions underlying this behavior, (*i.e.*, DNA–protein, protein–protein, or both) as well as the residues involved.

### Evidence supporting the formation of TDG condensates in cells

**Figure 5:**
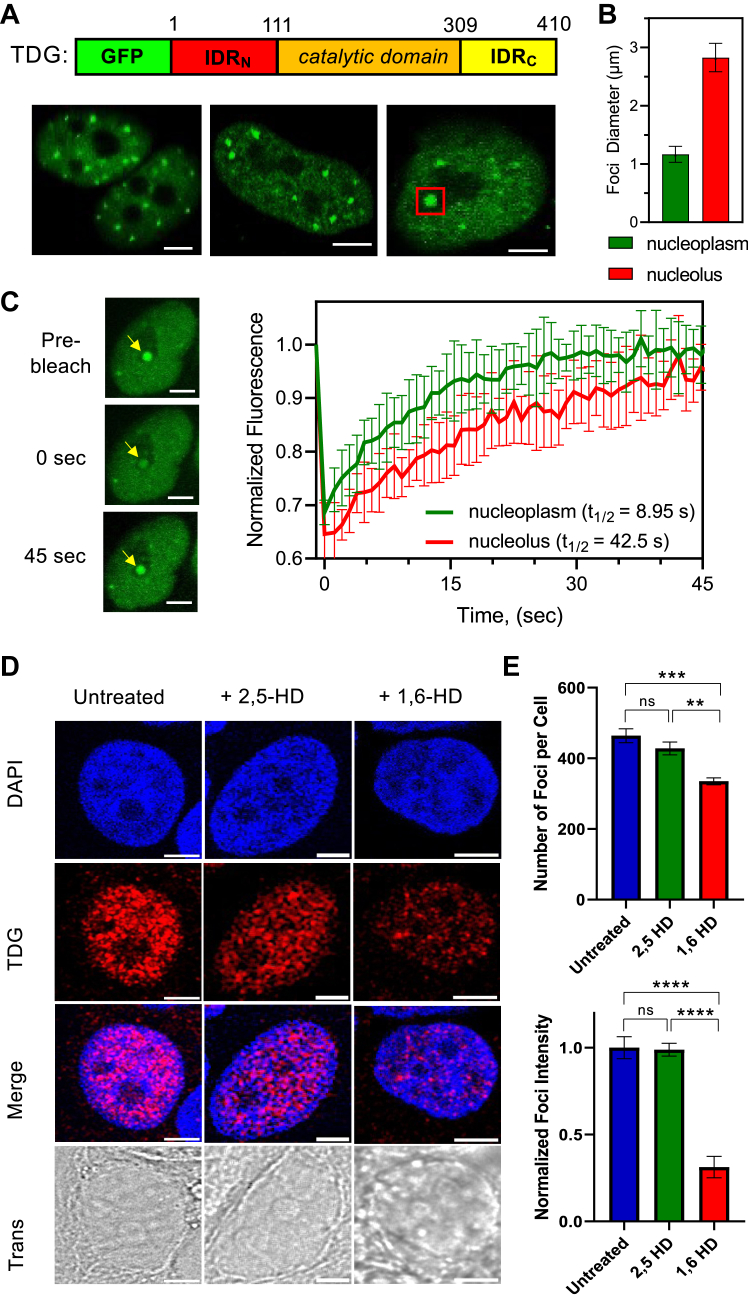
**TDG forms condensates in cells.***A*, representative confocal fluorescent microscopy images of HeLa cells transfected with TDG fused to GFP. *Red box* indicates a larger TDG condensate in the nucleolus. Scale bars represent 5 μm. *B*, diameter of individual chromatin droplets formed by TDG in live HeLa cells. Data are mean ± SD (nucleoplasm: n = 10 cells, nucleolus: n = 3 cells). *C*, confocal fluorescent microscopy images and FRAP curve of GFP-TDG in HeLa cells. Data are mean ± SD (nucleoplasm: n = 10 cells, nucleolus: n = 3 cells). Scale bars represent 5 μm. *D*, representative fluorescent microscopy images of endogenous TDG showing loss of TDG foci upon 1,6-HD treatment. TDG was detected by immunostaining-fixed cells with TDG-specific antibody. Scale bars represent 5 μm. *E*, quantification of foci number and intensity upon of TDG with either 2,5-HD or 1,6-HD. Foci number are mean ± SD (n = 3 cells) for pre- and post-10 min treatment. Foci intensity is mean ± SD for 464, 428, and 335 individual foci pre-, post-2,5-HD, and post-1,6-HD treatment, respectively. ∗∗*p* < 0.01; ∗∗∗*p* < 0.001; and ∗∗∗∗*p* < 0.0001. 1,6-HD, 1,6-hexanediol; 2,5-HD, 2,5-hexanediol; FRAP, fluorescence recovery after photobleaching; TDG, thymine DNA glycosylase.

We expanded our consideration of the biological relevance of TDG–chromatin condensates by examining the behavior of TDG in living cells. To this end, we transiently expressed GFP-tagged TDG (GFP-TDG) in HeLa cells and monitored its behavior using confocal fluorescence microscopy (Fig. 5*A*). We found that GFP-TDG localized to discrete nuclear puncta that ranged in size from 1 to 3 μm in diameter (Fig. 5*B*). In some cells (∼30%), puncta were observed in the nucleolus and were consistently larger than those distributed throughout the rest of the nucleus (Fig. 5*B*). The biological processes that regulate the size and localization of these structures was not immediately clear. Both sizes of nuclear puncta containing GFP-TDG met visual criteria of phase-separated condensates, including a spherical shape and rapid (<1 min) recovery after photobleaching (FRAP; Fig. 5*C*).

We next examined the phase behavior of endogenous TDG by immunostaining fixed MCF-7 cells with TDG-specific antibody. MCF-7 cells have robust TDG expression, and previous findings have shown that TDG plays a significant role in the cellular response of MCF-7 cells when exposed to estrogenic compounds, such as E2, making this an ideal cell line for our studies (20, 42, 43). In untransfected MCF-7 cells, endogenous TDG staining was observed in abundance and in a granular pattern throughout the nucleus, with an average of 450 distinct foci per nucleus (Fig. 5, *D* and *E*). siRNA knockdown of TDG significantly reduced the number of observable TDG foci, confirming that they were not a result of nonspecific antibody binding or aggregation (Fig. S11*A*). There was a moderate correlation between the nuclear distribution of TDG foci and 4′,6-diamidino-2-phenylindole staining (Pearson’s coefficient: 0.45 ± 0.08) (Fig. S11, *B* and *C*), suggesting the presence of chromatin within a large fraction of TDG foci as would be expected from our *in vitro* phase separation data. Exposure of MCF-7 cells to 1,6-HD resulted in considerable reduction in the number and signal intensity of TDG foci compared with untreated cells or cells treated with 2,5-HD (Fig. 5, *D* and *E*) (33, 34). Nearly identical behavior was observed for estrogen receptor α (ERα) and GATA3 (Fig. S12), two proteins known to form 1,6-HD-sensative liquid-like condensates in MCF-7 cells (34). Western blot analysis of 1,6-HD-treated cell lysates confirmed that TDG foci depletion occurred as a result of chemical disruption of the condensates and not changes in endogenous TDG levels (Fig. S13).

While these data provide evidence that TDG forms phase-separated condensates in the cell nucleus, we acknowledge the limitations of this preliminary study, including the use of overexpressed proteins and the caveats associated with FRAP and 1,6-HD data (44). Ultimately, additional experiments, including the demonstration of a critical concentration, will be needed to further support phase separation of TDG *in vivo.*

### DNA methylation modulates the phase behavior of TDG-mediated chromatin droplets

**Figure 6:**
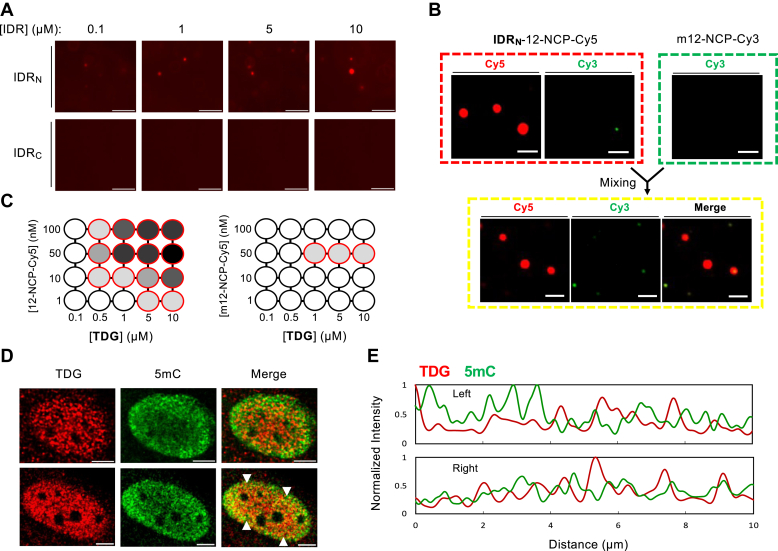
**DNA methylation regulates TDG-mediated chromatin phase separation.***A*, representative wide-field fluorescent microscopy images of mDNA_207_ (250 nM) in the presence of the indicated [IDR]. Scale bars represent 25 μm. *B*, methylated chromatin m12-NCP-Cy3 (50 nM) is unable to mix with preformed IDR_N_-12-NCP-Cy5 droplets generated by mixing 5 μM TDG with 12.5 nM chromatin. Similar data for IDR_C_-12-NCP-Cy5 droplets are presented in Fig. S15. Scale bars represent 5 μm. *C*, phase diagrams of TDG with unmethylated (12-NCP-Cy3) or methylated (m12-NCP-Cy3) chromatin under varying conditions. *Red circles* indicate phase separation. The *grayscale* indicates CV as described for Figure 3*E*. *D*, representative fluorescent microscopy images of endogenous TDG and 5mC as detected by immunostaining fixed cells with the corresponding antibody. Scale bars represent 5 μm. Pearson’s coefficient: 0.116 ± 0.04 (n = 15 cells). *E*, normalized fluorescence signal intensities of TDG (*red*) and 5mC (*green*) in the nuclei along the line (from *left* to *right*) indicated by *white arrowheads* in (*D*). 5mC, 5-methylcytosine; IDR, intrinsically disordered region; TDG, thymine DNA glycosylase.

We recently showed that DNA methylation inhibited the ability of TDG to convert soluble, monodisperse, chromatin fibers into oligomeric complexes that can be isolated *via* centrifugation-assisted precipitation (26). Therefore, we asked whether DNA methylation similarly affected the phase behavior of TDG with DNA and chromatin. Given that cytosine methylation impacts DNA flexibility, hydrophobicity, and hydration, it is not unexpected that it should impact the thermodynamic process of phase separation (45, 46, 47). For example, DNA methylation was shown to enhance methyl-CpG-binding protein 2 (MeCP2)–mediated phase separation of chromatin *in vitro* (48). We first examined whether individual IDRs of TDG could induce LLPS of a Widom 601-derived DNA fragment (207 bp) containing 19 methylated CpG dinucleotides (mDNA_207_). Methylation was carried out using the CpG methyltransferase M.ssSI, and complete methylation was validated by the methylation-sensitive restriction enzyme HpaII (Fig. S14). Whereas both TDG’s IDRs induced phase separation of the unmethylated 207 bp DNA (DNA_207_), only IDR_N_ induced phase separation of hypermethylated DNA (mDNA_207_) (Fig. 6*A*). IDR_C_ failed to induce phase separation of mDNA_207_ under any concentrations tested. These contrasting behaviors are further consistent with the notion that TDG’s IDRs interact with and condense chromatin through distinct modes, with the CTD being highly sensitive to DNA methylation. DNA methylation also influenced the dynamics of IDR–chromatin condensates. We found that preformed IDR–chromatin (12-NCP-Cy5) droplets were unable to mix with orthogonally labeled chromatin fibers (m12-NCP-Cy3) that were fully methylated at all CpG sites prior to reconstitution (Figs. 6*B* and S15). This is in stark contrast to our earlier observations that unmethylated chromatin can rapidly diffuse into and accumulate within the preformed IDR–chromatin condensates (Fig. 1, *F* and *G*). As with its individual IDRs, full-length TDG also induced chromatin condensation in a manner that was dependent on DNA methylation. Phase diagrams revealed that methylated chromatin fibers (m12-NCP-Cy3) severely impeded TDG-mediated droplet formation relative to unmethylated chromatin (12-NCP-Cy3) (Figs. 6*C* and S16). Together, these observations indicate that DNA methylation regulates the formation and coalescence of TDG-mediated condensates.

Finally, motivated by the apparent ability of DNA methylation to regulate the phase behavior of TDG–chromatin condensates *in vitro*, we examined the distribution of TDG foci relative to 5mC in MCF-7 nuclei using immunofluorescence staining. Because DNA methylation antagonizes TDG-mediated chromatin condensation *in vitro*, we expected endogenous TDG droplets to reside in nuclear compartments depleted of 5mC. Although immunofluorescence staining in MCF-7 cells did reveal some TDG foci depleted of 5mC (Fig. 6, *D* and *E*), no overall correlation was observed between the nuclear distribution of TDG foci and 5mC-dense regions (Pearson’s coefficient: 0.116 ± 0.04). Thus, it appears that the relationship between DNA methylation and TDG-mediated chromatin condensation is more complicated in cells, which could be dependent on specific genomic environments.

## Discussion

We have demonstrated that, in the presence of physiological salts and TDG concentrations, TDG has the intrinsic ability to assemble phase-separated condensates with DNA and chromatin *in vitro* and showed that the resulting chromatin droplets exhibit behaviors typical of phase-separated liquids. Evidence supporting this notion include (1) the formation of micrometer-sized droplets that exhibit spherical morphology, recovery rapidly after photobleaching, and can fuse; (2) condensation is sensitive to buffer conditions (*i.e.*, salt concentrations) and is reversible; (3) molecular exchange occurs between the dense and light phases; and (4) condensates are dissolved by 1,6-HD. The ability of TDG to induce chromatin phase separation and as our *in vitro* data suggest, LLPS provides a new and intriguing perspective on the mechanisms and functions of TDG and its role in associated genomic processes, such as transcription, DNA (de)methylation, and DNA repair.

We showed that TDG assembles chromatin condensates in a manner that is dependent on the unique properties of its terminal IDRs. This behavior is consistent with the notion that phase separation, as well as the material properties of the resulting condensates, is driven by multivalent interactions that depend on amino acid composition and sequence (1, 9). In the future, it will be important to characterize the underlying mechanisms of these interactions and to determine how the physical properties of these droplets are affected by various mutations related to disease. This behavior also suggests that post-translational modifications, which occur extensively within TDG’s IDRs, will play a role in modulating the ability of TDG to undergo LLPS. For example, several lysine residues within TDG’s IDR_N_ can be acetylated, resulting in the neutralization of positive charges that are important for DNA binding and likely contribute to multivalent interactions that promote chromatin condensation (49, 50, 51, 52). SUMOylation of TDG’s IDR_C_ is also expected to impact its phase behavior (53, 54, 55). Interestingly, SUMOylation has been shown to regulate the translocation of TDG into promyelocytic leukemia (PML) nuclear bodies (NBs) through a poorly understood mechanism (55). Given that the formation and structure of PML NBs has been proposed to involve LLPS (56), it is tempting to speculate that modulation of TDG’s phase-separation behavior by SUMOylation regulates its translocation into PML NBs. Thus, phase separation may play an important role in directing the subcellular localization of TDG. Finally, TDG’s IDR_N_ has been shown to be important for proper substrate binding and catalysis. For example, residues 82 to 110 impart tight DNA binding to allow processing of less-favorable G•T mismatches, although at the expense of enzyme turnover (22, 54). Residues 51 to 111 of IDR_N_ have also been shown to interact with the catalytic domain, leading to the proposal that IDR_N_ regulates TDG’s substrate specificity and catalytic activity through an allosteric mechanism (23). Given the contributions of IDR_N_ to lesion processing, the involvement of these same residues in inducing chromatin phase separation suggests a link between the two processes. Thus, it will be important to determine the impact of phase separation on TDG’s catalytic activity in the future.

TDG is known to directly interact with numerous proteins, and these interactions often occur through its terminal IDRs (16). A phase-separation model readily explains how such diverse interactions can occur through these low-complexity domains. Indeed, several TDG-binding partners, including ERα (6), p300 (57), SRC-1 (58), and RARα (34), contain IDRs and have been shown to undergo LLPS. Notably, in response to E2, TDG is recruited to active enhancers that also recruit several of its binding partners (*e.g.*, ERα and p300) and other IDR-containing transcription (co)factors (20). Recent evidence suggests that the accumulation of these proteins at E2-responsive enhancers results in the formation of liquid-like phase-separated condensates, which subsequently drive long-range genomic interactions through coalescence of different condensates (34). Interestingly, at a subset of E2-responsive enhancers, depletion of TDG has been shown to disrupt long-range genomic interactions and transcription of the corresponding genes, implicating TDG in these processes (20). Considering the data presented herein, one possible explanation is that TDG is required for the assembly, stability, and/or coalescence of biological condensates at these enhancers.

The implications of this work also extend to active DNA demethylation, a process that is closely associated with transcriptional activation (14, 15). Numerous gene promoters and enhancers undergo demethylation during transcriptional activation and, in some instances, demethylation is linked to chromosomal rearrangements (18, 19, 42, 43, 59). LLPS has been shown to play a crucial role in chromatin organization and gene transcription (4, 6). Therefore, given the intimate relationship of DNA demethylation (and TDG) with both processes, along with the involvement of numerous IDR-containing proteins, it is not unreasonable to predict that demethylation involves the formation of biomolecular condensates, possibly *via* LLPS. Indeed, DNA demethylation requires the coordinated recruitment of various transcription (co)factors, histone modifiers, and BER proteins, often in a TDG-dependent manner. LLPS offers a potential mechanism to rapidly assembly, organize, and disassemble high concentrations of these factors in a spatiotemporal manner. In the light of the work presented, the concept of LLPS to target and coordinate demethylation activities, potentially through TDG, is appealing. Ultimately, further cellular and genomic investigations are required to establish functional relationships between DNA (de)methylation and TDG’s ability to assemble chromatin condensates.

Finally, to the best of our knowledge, this is the first report of a DNA glycosylase assembling phase-separated chromatin condensates, which has important implications for BER. For example, a phase separation (or LLPS) model for BER is consistent with the known coupling of this pathway to transcription and provides an attractive mechanism for assembly of so-called “BERosomes” at sites of DNA damage and/or chemical modification (60, 61, 62, 63). This model is also supported by the observations that key proteins involved in BER, including several glycosylases (*e.g.*, TDG and NEILs), APE1, and XRCC1, contain one or more IDRs (Fig. S17). Indeed, a recent study demonstrated that APE1 assembles liquid-like phase-separated condensates *in vitro* in a manner dependent on its IDR (64). In the future, it will be important to further evaluate the phase behavior of these and other BER-associated factors, as well as their influence on the assembly and properties of phase-separated condensates mediated by TDG.

## Experimental procedures

### Reagents

All restriction enzymes (PfIMI, BstXI, and HpaII), CpG methyltransferase (M.SsI), and Phusion High-Fidelity DNA Polymerase were purchased from New England Biolabs. Maleimide (catalog nos.: 21380 and 23380) and *N*-hydroxysuccinimide (catalog no.: 23320) ester–modified Cy3 and Cy5 dyes were purchased from Lumiprobe Life Science Solutions. Recombinant human histone H4.1 was purchased from the histone source. All synthetic oligonucleotides were purchased from Integrated DNA Technologies. The GenCatch Advanced PCR Extraction kit (catalog no.: 23-60250) was acquired from Epoch Life Science. Sigmacote (catalog no.: SL2-25ML) and poly-l-lysine (catalog no.: P9155) were both purchased from Sigma–Aldrich. HeLa and MCF-7 cells were obtained from American Type Culture Collection. Phenol red–containing Dulbecco's modified Eagle’s medium (DMEM) (catalog no.: 11995-065), reduced serum media, Opti-MEM (catalog no.: 31985-070), Lipofectamine 2000 (catalog no.: 11668030), 10% fetal bovine serum (FBS) (catalog no.: A4766801), and Lipofectamine RNAiMAX (catalog no.: 13778100) were purchased from Thermo Fisher Scientific. Streptomycin and Triton X-100 (catalog no.: 9002-93-1) were purchased from Sigma–Aldrich. The expression vector for GFP-TDG (catalog no.: HG13000-ANG) was purchased from Sino Biological. The 48-well glass bottom plates (catalog no.: P48G-1.5-6-F) were purchased from MatTek. The 96-well cell culture microplates (catalog no.: 655180) and the 24-well cell culture microplates (catalog no.: 662160) were both purchased from Greiner Bio-One. The 8-well 15 μ-Slides (catalog no.: 80826) were purchased from Ibidi. The ON-TARGET plus SMART pool siTDG (catalog no.: L-003780-01-0005) was obtained from Dharmacon. The PBS (catalog no.: 46-013-CM) was obtained from Corning. Bovine serum albumin (BSA) (catalog no.: 0332-100G) for blocking was obtained from VWR. The rrabbit anti-TDG antibody (catalog no.: A304-365A) was purchased from Bethyl Laboratories. The goat anti-Rabbit immunoglobulin G (IgG) (H + L) highly cross-adsorbed secondary antibody, Alexa Fluor Plus 647 (catalog no.: A32733), goat antimouse IgG (H + L) cross-adsorbed secondary antibody, Cyanine3 (catalog no.: A10521), and Hoechst 33342, trihydrochloride, trihydrate for nuclei staining (catalog no.: H3570) were purchased from Invitrogen. The mouse anti-Erα antibody (catalog no.: ab93021), mouse anti-5mC primary antibody (catalog no.: ab10805), and goat antimouse IgG H&L (Alexa Fluor 555) secondary antibody (catalog no.: ab150114) were purchased from Abcam. The mouse anti-GATA3 (HG3-31) antibody (catalog no.: sc-268) was purchased from Santa Cruz Biotechnology.

### Methods

#### Histone preparation and octamer refolding

Recombinant human histones H2A_N11OC_, H2A.1, H2B.1, and H3.1 were expressed and purified using our previously described methods (26, 27). Histone H2A_N11OC_ was fluorescently labeled using maleimide Cy3 and Cy5 dyes as instructed by the manufacturer. The purified histone octamers were refolded and purified following established protocols (27, 65) and stored in octamer buffer (2 M NaCl, 5 mM β-mercaptoethanol, 0.2 mM phenylmethylsulfonyl fluoride (PMSF), 10 mM Hepes, pH 7.8) at 4 °C until further use.

#### TDG expression and purification

Full-length human TDG and truncated TDG variants used herein were expressed and purified as described previously (66, 67). Purified proteins were stored at −80 °C in HP50 buffer (50 mM Hepes, pH 8, 50 mM NaCl, 10% glycerol, 10 mM β-mercaptoethanol, and 1 mM PMSF) until use.

#### Preparation of DNA templates

The DNA template used to assemble 12-NCP-Cy3/Cy5 nucleosome arrays, referred to as 12-DNA, consisted of 12 copies of the “Widom 601” positioning sequence separated by 30 bp of linker DNA (Fig. S2*A*). The DNA sequence and assembly of 12-DNA has been reported previously (26, 27). The DNA used to produce TFF1e-Cy3/Cy5 chromatin, referred to as TFF1e-DNA, was generated by PCR amplification of 150 ng of human genomic DNA using primers TFF1eFWD and TFF1eREV (Table S1) employing Phusion High-Fidelity DNA Polymerase according to the manufacturer’s instructions. Following PCR amplification, TFF1e-DNA was purified and desalted using the GenCatch Advanced PCR Extraction kit (Fig. S6*A*). DNA_207_ was prepared by PCR amplification of 601 DNA with primers TET2_FWD_PfIMI and BstXI.REV (Table S2). Following PCR amplification, DNA_207_ was purified and desalted using the GenCatch Advanced PCR Extraction kit.

#### Reconstitution of nucleosome arrays

Nucleosome arrays (12-NCP-cy3/5 and TFF1e-NCP-cy3/5) were reconstituted *via* slow salt dialysis as before (26, 27) using the corresponding DNA (12-DNA and TFF1e-DNA, respectively) and histone octamers described previously. Samples were centrifuged immediately after salt dialysis at 13,000× RPM for 20 min at 4 °C. The soluble chromatin substrates were collected and stored at 4 °C in buffer NB (25 mM NaCl, 0.1 mM PMSF, 10 mM Hepes, pH 7.8), until use. Reconstituted arrays were analyzed by 0.6% agarose gel electrophoresis (Figs. S2*B*, S5*B* and S14*C*) to confirm the absence of free DNA.

#### Nucleosome occupancy assay

To confirm nucleosome saturation of arrays, ∼150 ng of array (or the corresponding free DNA) was digested with 7.5 units of BstXI and PfIMI restriction enzymes in buffer NB supplemented with 2 mM MgCl_2_. Both sets of samples (naked DNA and arrays) were analyzed side by side on a 5% native PAGE (59:1 acrylamide:bisacrylamide) (Fig. S2*C*). Prior to loading onto the gel, the final glycerol concentration of the samples was adjusted to 5%, using buffer NB supplemented with 30% glycerol. Following digestions, the absence of free 610 DNA (<1%) and the presence of a nucleosome band confirm full nucleosome occupancy in array samples.

#### M.SssI methylation of DNA and nucleosome arrays

For the methylation of the 12-DNA and DNA_207_, ∼10 μg of DNA was incubated with 10 units of M.SssI in 1× CutSmart buffer supplemented with 0.4 mM SAM at 37 °C for 4 h. The reactions were heat inactivated *via* incubation at 70 °C for 20 min and ethanol precipitated. To confirm successful methylation at CpG sites, a 75 fmol aliquot was digested with 10 units of HpaII in a 10 μl solution containing 1× CutSmart buffer at 37 °C for a total of 45 min. After digestions, glycerol was added (5%, v:v), and the sample, along with undigested controls, was analyzed side by side *via* agarose gel electrophoresis (0.7% for m12-NCP and 1% for mDNA) (Fig. S14, *A* and *B*). HpaII-resistant 12-DNA was used in subsequent nucleosome array reconstitutions and confirmed to form chromatin *via* native agarose gel electrophoresis (Fig. S14, *C* and *D*).

#### *In vitro* phase separation assay

Phase separation experiments were conducted by combining TDG (or its truncations) with fluorescently labeled DNA or nucleosome arrays at the indicated concentration in 1× LLPS buffer (10 mM Hepes, 100 mM KCl, 1 mM MgCl_2,_ PEG 8 K). Unless stated otherwise, droplet formation by TDG’s IDRs was carried out in the presence of 5% PEG, whereas droplet formation by full-length TDG or its truncated variants was carried out in the presence of 1% PEG. Reactions were prepared by mixing 1:1 volumes of each components at 2× their intended concentration in 1× LLPS buffer. Samples were allowed to incubate for 30 min before transferring to a coverslip for imaging (later). Prior to use, coverslips were siliconized using Sigmacote as directed by the manufacturer. The presence of TDG within the condensates was confirmed by immunofluorescence staining. Following droplet formation, the suspension was mixed with 1 μl of solution containing a 1:2000 dilution of rabbit anti-TDG antibody (α-TDG_360–410_; catalog no.: A304-365A) and the goat anti-rabbit IgG (H + L) secondary antibody conjugated with Alexa Fluor Plus 647 (catalog no.: A32733) in 1× LLPS buffer at room temperature for 10 min prior to transferring to a glass coverslip for imaging.

#### Confocal microscopy

Fluorescence confocal imaging was performed on an Olympus FV1000 laser-scanning confocal microscope using a 60× oil-immersion objective (Plan-Apochromatic, numerical aperture: 1.4) and the FluoView-10 (version 3.1) acquisition software to capture both fluorescent and brightfield images. A transmitted-light photomultiplier detector was used to acquire transmitted light images concurrently with the fluorescence images. For static droplets on coverslips, images acquired on the Cy3 channel were obtained using a 543 nm laser excitation wavelength and a 555 to 625 nm emission (monochromator) or the Cy5 channel using a 635 nm laser excitation wavelength and 655 to 755 nm emission (band-pass filter). For droplet FRAP experiments, five frames were acquired prior to photobleaching to determine baseline fluorescence, and then droplets were bleached at a single point by pulsing the laser 20 times at 100% transmissivity with a dwell time of 8 μs. Recovery was recorded in time lapse at a rate of 2 to 15 s between frames, which was varied depending on the rate of recovery.

Fluorescence images of fixed cells were acquired using the same confocal instrument described previously or by using a Leica SP8 confocal microscope. For the Olympus, images were acquired at the following excitation/emission wavelengths: 405 nm/410 to 510 nm (monochromator), 543 nm/555 to 625 nm (monochromator), and 635 nm/655 to 755 nm (band-pass filter). FRAP experiments on nuclear foci were performed at an excitation of 488 nm and emission range of 500 to 600 nm (monochromator). An initial five frames were acquired on the cell to determine baseline fluorescence. Bleaching was performed by focusing the laser at the center of the puncta and pulsing at 100% laser power for 20 to 60 pulses. Recovery was recorded in time lapse at a rate of ∼3.4 frames per second for 300 frames. FRAP measurements were fitted with a two-phase exponential, and half time of recovery was determined graphically for *in vitro* and *in vivo* experiments (2). For the Leica SP8, fluorescent and brightfield images were acquired using an HC PL APO 40×/1.10 W motCORR CS2 water immersion objective in conjunction with a 405 nm CW laser and a 470 to 670 nm white pulsed laser. A standard PMT detector was used for detection, and all images were acquired using the Leica Application Suite X (version 5.0.2).

#### Droplet mixing experiments

Preformed droplets were prepared by mixing 5 μM IDR_N_ or IDR_C_ with 12.5 nM 12-NCP-Cy5/Cy3 in 1× LLPS buffer as described previously. After 10 min, droplet solutions were then rapidly mixed with one-fourth volume of either PI (diluted 1:2500 from stock) or fluorescent nucleosome arrays (50 nM) in 1× LLPS buffer by pipetting up and down and then transferring to a glass coverslip for imaging as described previously.

#### Droplet reversibility assays

The ability of preformed TDG–chromatin condensates to withstand a range of salt (NaCl) and 1,6-HD concentrations was assessed by combing 3 μl suspension of preformed TDG–chromatin droplet with 1 μl of NaCl or 1,6-HD prepared at 4× the desired concentration. All solutions were in 1× LLPS buffer. Mixtures were incubated for 5 min at room temperature before being transferred to a Sigmacote-treated glass coverslip for imaging as described previously.

#### Generation of phase diagrams

Different concentrations of TDG (or its truncations) were titrated against a concentration gradient of 12-NCP-Cy3 chromatin (or m12-NCP-Cy3) in 1× LLPS buffer containing 1% PEG and imaged at the glass bottom of a 96-well plate as described previously.

#### Cell culture

HeLa cells were cultured in phenol red–containing DMEM supplemented with 10 mM Hepes, 1 mM GlutaMax, 100 U/ml penicillin, 0.1 mg/ml streptomycin, and 10% FBS. MCF-7 cells were cultured identically, except in phenol red–free DMEM containing only 5% FBS. One hour prior to imaging, MCF-7 cells were treated with 100 nM E2. All cells were maintained at 37 °C in a humidified CO_2_ (5%) atmosphere. All glass and plastic surfaces used were pretreated with 0.01% poly-l-lysine before use.

#### TDG overexpression and knockdown

The expression vector for GFP-TDG (Sino Biological; catalog no.: HG13000-ANG) was transfected into HeLa cells using Lipofectamine 2000 according to the manufacturer’s instructions. Briefly, DNA–lipid complexes consisting of 500 ng plasmid were formed for 20 min in in reduced serum medium (Opti-MEM) at room temperature and then added to 24-well plates containing 1 × 10^4^ HeLa cells under 0.45 ml DMEM. The media were replaced with fresh DMEM after 8 h, and cells were grown for an additional 48 h before being transferred to 48-well glass bottom plates. Cells were imaged 18 to 24 h later in phenol red–free DMEM.

For TDG knockdown experiments, MCF-7 cells were reverse transfected with 25 nM ON-TARGET plus SMART pool siTDG using Lipofectamine RNAiMAX according to the manufacturer’s instructions. After 14 h, the media were replaced with fresh phenol red–free DMEM, and cells were grown for an additional 48 h before being transferred to an 8-well 15 μ-Slide for imaging.

#### Immunofluorescence imaging of endogenous TDG

MCF-7 cells were treated with 100 mM E2 for 1 h before being fixed with 4% formaldehyde solution in PBS for 15 min at 37 °C. Cells were permeabilized with PBS containing 0.2% (v/v) Triton X-100 (PBS-T) for 10 min at 37 °C. Following fixation and permeabilization, the cells were blocked with 3% BSA in PBS-T at 37 °C for 30 min followed by several washes with PBS-T. The cells were incubated with a rabbit anti-TDG antibody (α-TDG_360–410_; 150-fold dilution in PBS-T) for 1 h at 37 °C. After washing with PBS-T three times, cells were treated with an Alexa-647 conjugated goat anti-rabbit IgG (H + L) secondary antibody conjugated with an Alexa Fluor Plus 647 (1000-fold dilution in PBS-T) for 30 min at 37 °C and imaged as described previously. For Erα and GATA3 immunostaining, cells were incubated with either mouse anti-Erα antibody (20-fold dilution in PBS-T) or mouse anti-GATA3 antibody (200-fold dilution in PBS-T) for 1 h at 37 °C. This was followed by treatment with Cy3-conjugated goat antimouse IgG H&L (Alexa Fluor 555) secondary antibody (1000-fold dilution in PBS-T) for 30 min at 37 °C. Nuclei were stained with Hoechst 33342 for 10 min at 37 °C prior to imaging.

For combined TDG and 5-mC immunostaining, a slightly modified approach was used. Cell fixation was performed with 4% formaldehyde in PBS for 30 min at room temperature, and permeabilization was carried out using 1% BSA in PBS-T for 4 min at room temperature. The permeabilization solution was replaced with ice-cold 88% methanol in PBS and allowed to incubate at room temperature for 5 min. The cells were then washed twice with the same permeabilization buffer and then treated with 2 M HCl for 30 min at 37 °C. The HCl was replaced with 0.1 M sodium borate buffer (pH 8.5) and incubated for 5 min at room temperature. All subsequent steps were conducted as before. For visualizing 5-methylcytosine (5mC), the mouse anti-5mC primary antibody (60-fold dilution in PBS-T) and goat antimouse IgG H&L secondary antibody conjugated with Alexa Fluor 555 (100-fold dilution in PBS-T) was used.

#### Data and statistical analyses

All data analyses were conducted in ImageJ (version 1.53c). Unless stated otherwise, all images were acquired under identical microscopy settings for a given experiment. Equivalent brightness and contrast (scaled linearly) were used when depicting microscopy images in a given panel. For droplet diameter measurements, particles >0.5 μm^2^ were included in the analysis. For *in vitro* droplet FRAP experiments, the intensity of the photobleached region was normalized to the fluorescence of the entire droplet at each respective time point. For phase diagrams, images (.tif) were baseline corrected, and the mean pixel intensity and standard deviation measurements were calculated using FIJI (ImageJ2) software (version 2.3.0/1.53q) and used to determine the CV. Foci number and relative intensity of TDG, Erα, and GATA3 in fixed cells following 1,6-HD or 2,5-HD treatment were determined using the particle analysis tool in FIJI. GFP-TDG FRAP experiments were quantified by normalizing the fluorescent intensity of a fixed area surrounding the FRAP-targeted foci to the rest of the nuclei’s fluorescence and then plotted with respect to a pre-FRAP reading. Colocalization analysis on TDG and 5mC was preformed using Coloc2 on FIJI (ImageJ2) software (version 2.3.0/1.53q). Histograms of both channels were produced using the plot profile function on FIJI.

All statistical analyses were done on GraphPad Prism (version 8.4.2) and presented as means and standard deviations. Datasets within a given experiment were compared using unpaired one-way ANOVA. Then Tukey’s multiple comparisons test (α = 0.05) was used for comparing significant differences between each condition tested.

## Data availability

The data generated during all experiments are available from the author upon reasonable request.

## Supporting information

This article contains supporting information (68).

## Conflict of interest

The authors declare that they have no conflicts of interest with the contents of this article.
