# Supplementary material for: Thymine DNA glycosylase mediates chromatin phase separation in a DNA methylation–dependent manner
Source: J Biol Chem. 2023 Jun 10;299(7):104907. doi: 10.1016/j.jbc.2023.104907 (PMC10404674; doi:10.1016/j.jbc.2023.104907)
Supplement: Supporting information [file mmc1.pdf]

Supporting Information

Thymine DNA Glycosylase Mediates Chromatin Liquid-Liquid Phase Separation in a DNA Methylation-Dependent Manner

Lauren A. McGregor[1], Charles E. Deckard III[2], Justin A. Smolen[1], Gabriela M. Porter[1], and Jonathan T. Sczepanski[1,*]

[1] Department of Chemistry, Texas A&M University, College Station, Texas, 77843, USA

[2] Current address: TRACTION-MD Anderson Cancer Center, Houston, Texas, 77054, USA

[*] To whom correspondence should be addressed. Email: jon.sczepanski@chem.tamu.edu

S1. Supplementary Figures

```
        10         20         30         40         50         60
MEAENAGSYS LQQAQAFYTF PFQQLMAEAP NMAVVNEQQM PEEVPAPAPA QEPVQEAPKG

        70         80         90        100        110        120
RKRKPRTTEP KQPVEPKKPV ESKKSGKSAK SKEKQEKITD TFKVKRKVDR FNGVSEAELL

       130        140        150        160        170        180
TKTLPDILTF NLDIVIIGIN PGLMAAYKGH HYPGPGNHFW KCLFMSGLSE VQLNHMDDHT

       190        200        210        220        230        240
LPGKYGIGFT NMVERTTPGS KDLSSKEFRE GGRILVQKLQ KYQPRIAVFN GKCIYEIFSK

       250        260        270        280        290        300
EVFGVKVKNL EFGLQPHKIP DTETLCYVMP SSSARCAQFP RAQDKVHYYI KLKDLRDQLK

       310        320        330        340        350        360
GIERNMDVQE VQYTFDLQLA QEDAKKMAVK EEKYDPGYEA AYGGAYGENP CSSEPCGFSS

       370        380        390        400        410
NGLIESVELR GESAFSGIPN GQWMTQSFTD QIPSFSNHCG TQEQEEESHA
```

IDR_N
IDR_C

Figure S1: Amino acid sequence of thymine DNA glycosylase (TDG) from *Homo sapiens*. TDG's intrinsically disordered, terminal domains; IDR_N and IDR_C, are highlighted in yellow and cyan, respectively. Charged and polar amino acids are denoted by bolded and underlined amino acid letters.

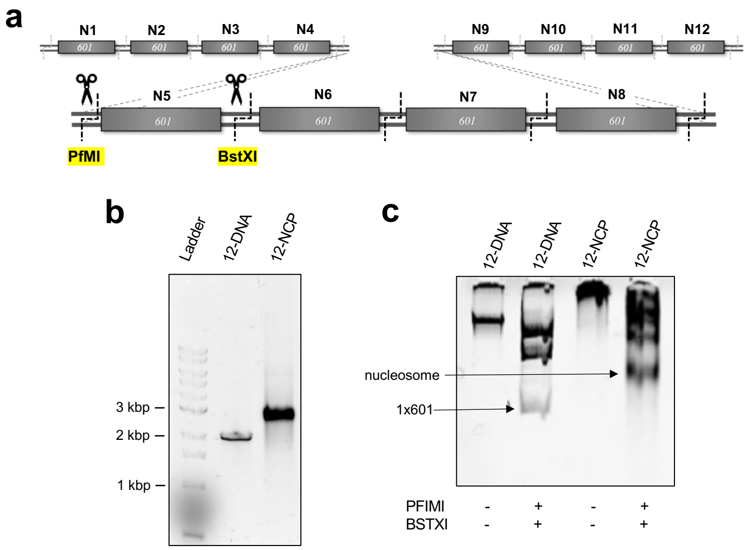

Figure S2. Preparation of 12-NCP-Cy3/Cy5 chromatin and confirmation of nucleosome occupancy. (a) The DNA template used to assemble 12-NCP-Cy3/Cy5 chromatin (referred to herein as 12-DNA) consists of 12 copies of the "Widom 601" nucleosome positioning sequence, each of which is separated by 30 bp of linker DNA. (b) Representative native gel analysis (0.6% agarose) of 12-NCP-Cy3 arrays reconstituted with 1.1 molar equivalent of Cy3-labeled histone octamer. (c) Representative restriction digestion analysis (5% native PAGE, 59:1 acrylamide:bisacrylamide) of 12-DNA and 12-NCP-Cy3 demonstrating complete nucleosome occupancy.

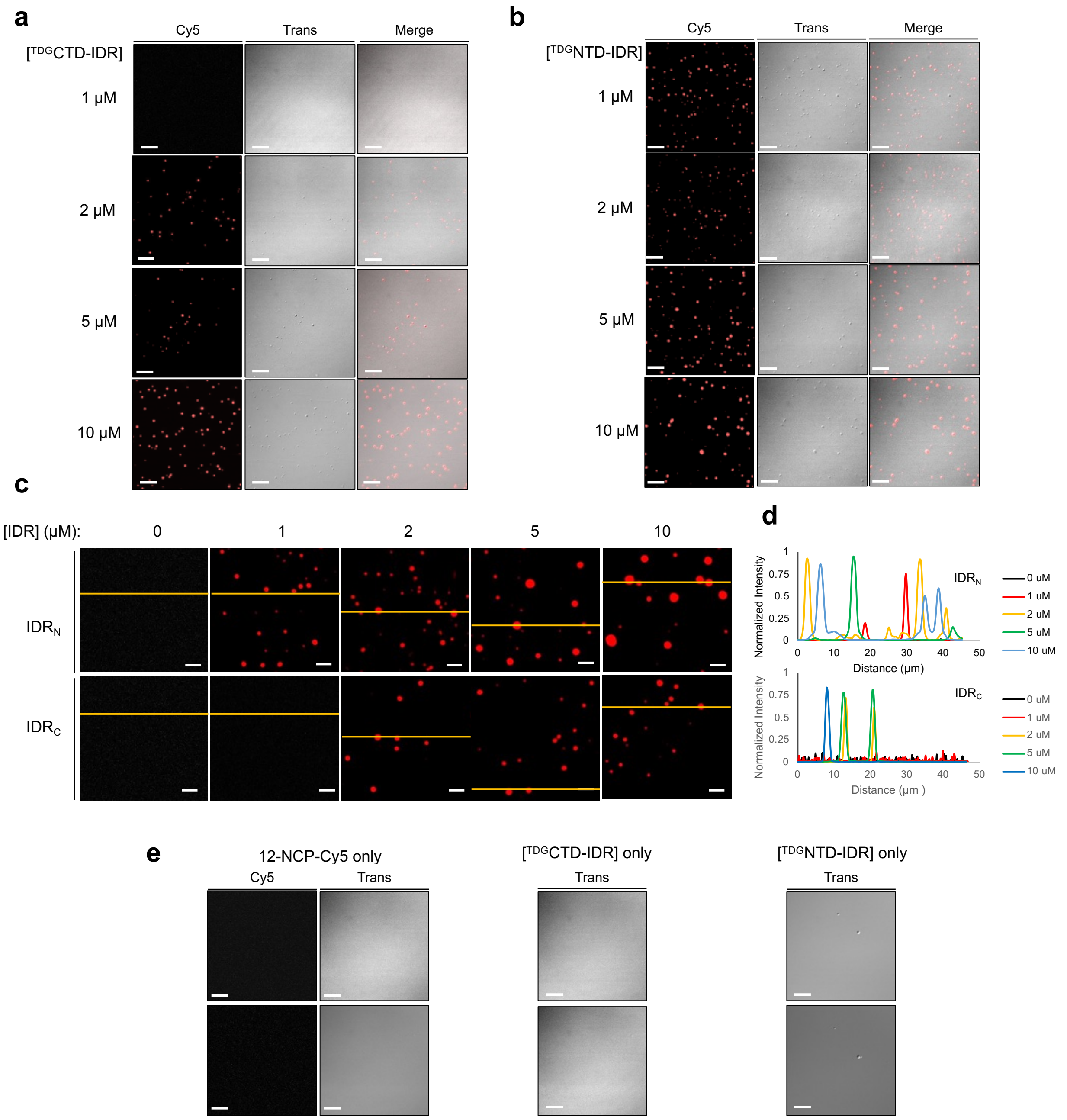

Figure S3. TDG's IDRs induce chromatin condensation. (a,b) Representative confocal microscopy images of chromatin droplets formed by the indicated concentration of either IDR_N (a) or IDR_C (b) in the presence of 25 nM 12-NCP-Cy5. (c) Representative fluorescent confocal microscopy images of 12-NCP-Cy5 chromatin (25 nM) in the presence of the indicated [IDR]. Scale bars: 5 μm. These images are reused from Figure 1b and were positioned here to allow for the convenient visualization of the source data for panel S3d. (d) Fluorescence intensity profile of 12-NCP-Cy5 chromatin along the yellow lines in panel (c). (e) Representative confocal microscopy images of 12-NCP-Cy5 (25 nM) and TDG's IDRs (5 μM) in isolation. Scale bars: 10 μm.

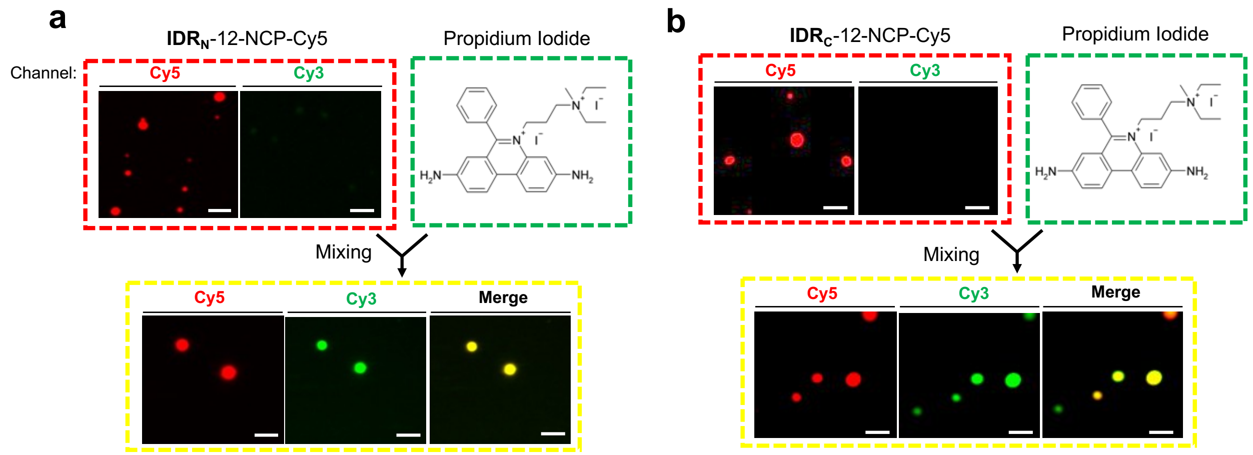

Figure S4. Representative confocal fluorescent microscopy images demonstrating that propidium iodine penetrates into preformed IDR_N-12-NCP-Cy5 (a) and IDR_C-12-NCP-Cy5 (b) droplets generated by mixing 5 µM of the IDR with 12.5 nM chromatin. All scale bars: 5 µm.

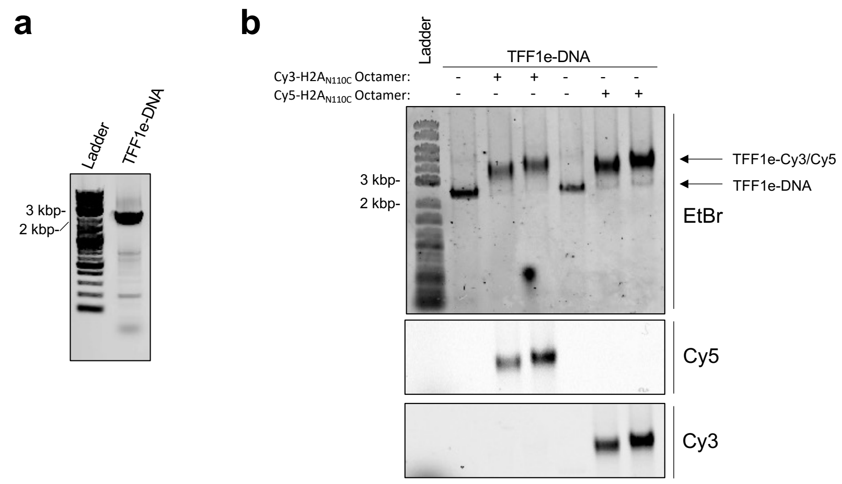

Figure S5. Preparation of TFF1e chromatin. (a) Native gel analysis (0.6% agarose) of TFF1e-DNA generated from human genomic DNA using TFF1eFWD and TFF1eREV (Table S1). (b) Native gel analysis (0.6% agarose) of TFF1e-Cy3/Cy5 chromatin reconstituted with 1.3 molar equivalent histone octamer containing Cy3/Cy5-labeled H2A$_{N110C}$.

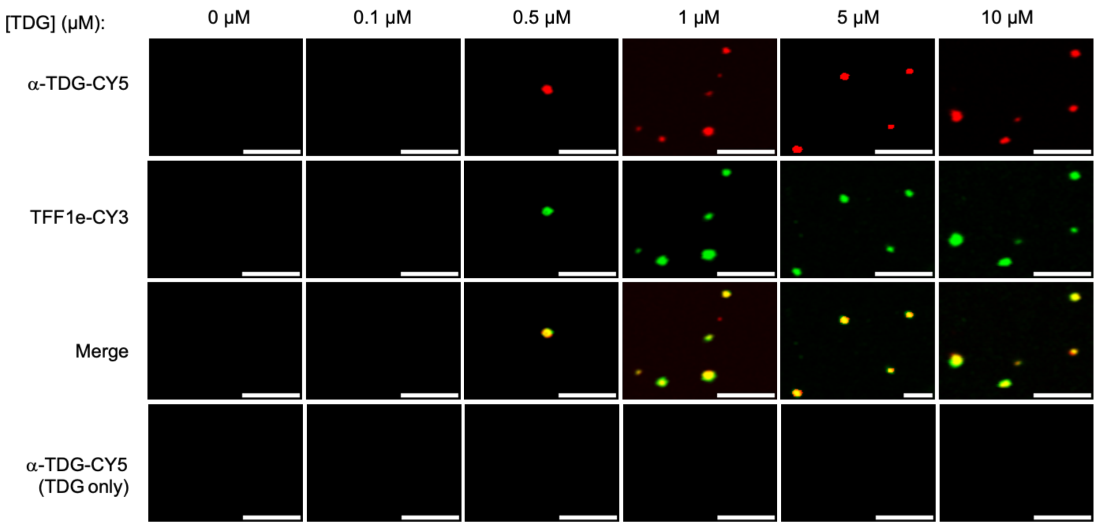

Figure S6. Confocal microscopy images of 25 nM TFF1e-Cy3 chromatin with increasing amounts of TDG in 1× LLPS buffer containing 1% PEG. Colocalization of TDG into the droplets was visualized by *in situ* immuno-staining using Cy5-label anti-TDG antibody (α-$TDG_{360\text{-}410}$). No staining was observed with α-TDG-Cy5 in the absence of TFF1-Cy3 chromatin. All scale bar: 10 μm. The images in the "1 μM" column were reused from Figure 3c and were positioned here to allow for the convenient visual comparison to other images in this series.

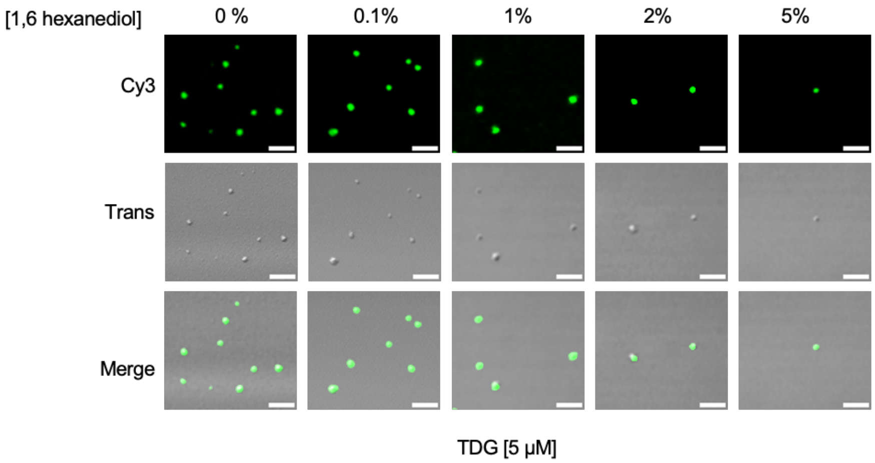

Figure S7. Representative confocal microscopy images of TFF1e-NCP-Cy3 condensates generated using 25 nM of TFF1e-NCP-Cy3 and 5 μM TDG in 1× LLPS buffer followed by incubation with 0 – 5% 1,6-HD. Images were taken 5 min after 1,6-HD treatment. All scale bar: 5 μm.

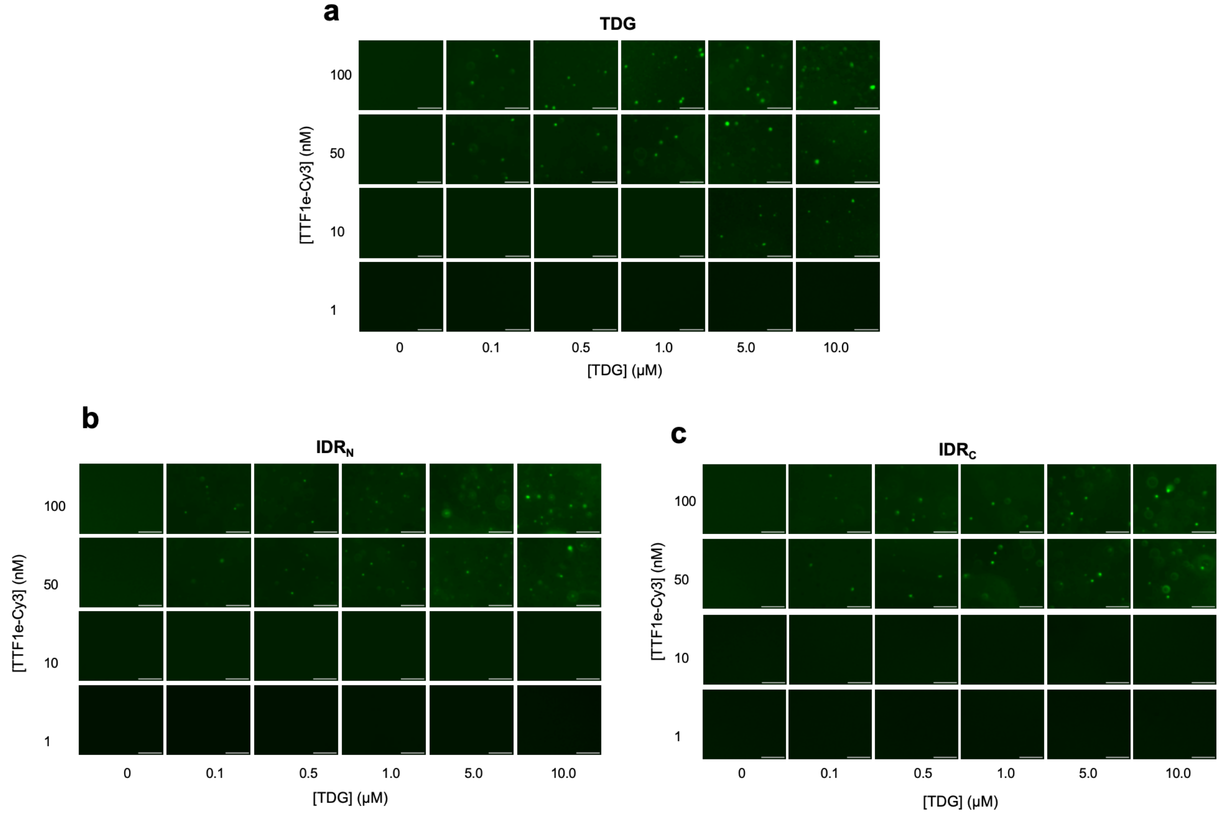

Figure S8. Representative wide-field fluorescent microscopy images used to generate phase diagrams (Figure 3e). The indicated concentrations of TDG (a), IDR$_N$ (b), or IDR$_C$ (c) were titrated against a concentration gradient of TFF1e-Cy3 chromatin in 1× LLPS buffer containing 1% PEG and imaged at the glass bottom of a 96-well plate. The images in the top row of Figure S8a are reused from Figure 3a and were positioned here to allow for the convenient visual comparison to other images in this series. All scale bar: 25 μm.

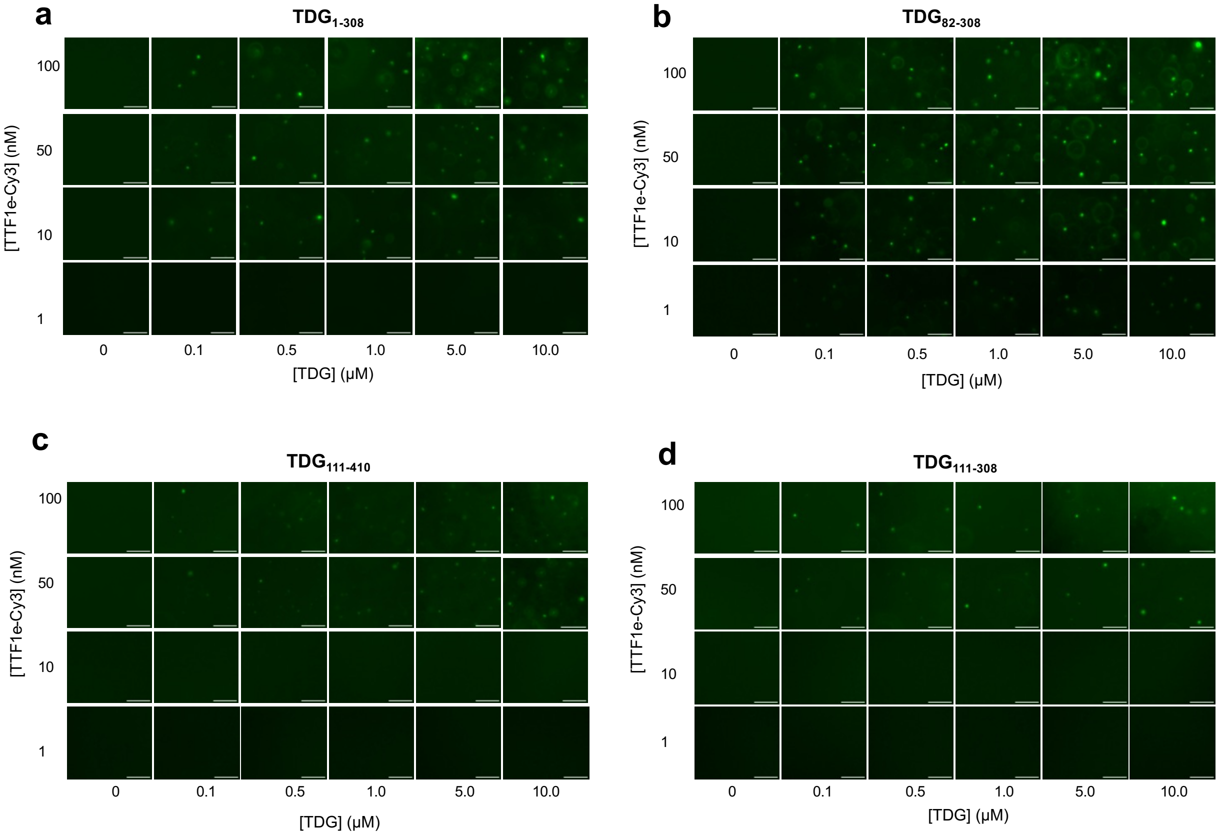

Figure S9. Representative wide-field fluorescent microscopy images used to generate phase diagrams for TDG truncations (Figure 4). The indicated concentrations of $TDG_{1\text{-}308}$ (a), $TDG_{82\text{-}308}$ (b), $TDG_{111\text{-}410}$ (c), or $TDG_{111\text{-}308}$ (d) were titrated against a concentration gradient of TFF1e-Cy3 chromatin in 1× LLPS buffer containing 1% PEG and imaged at the glass bottom of a 96-well plate. All scale bar: 25 µm.

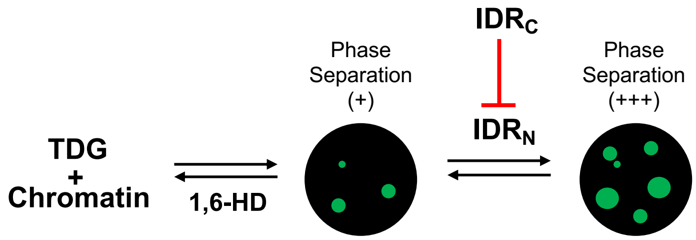

Figure S10. TDG induces reversible chromatin phase separation in a manner that is dependent on it IDRs. Whereas IDR$_N$ promotes phase separation, IDR$_C$ antagonizes this process.

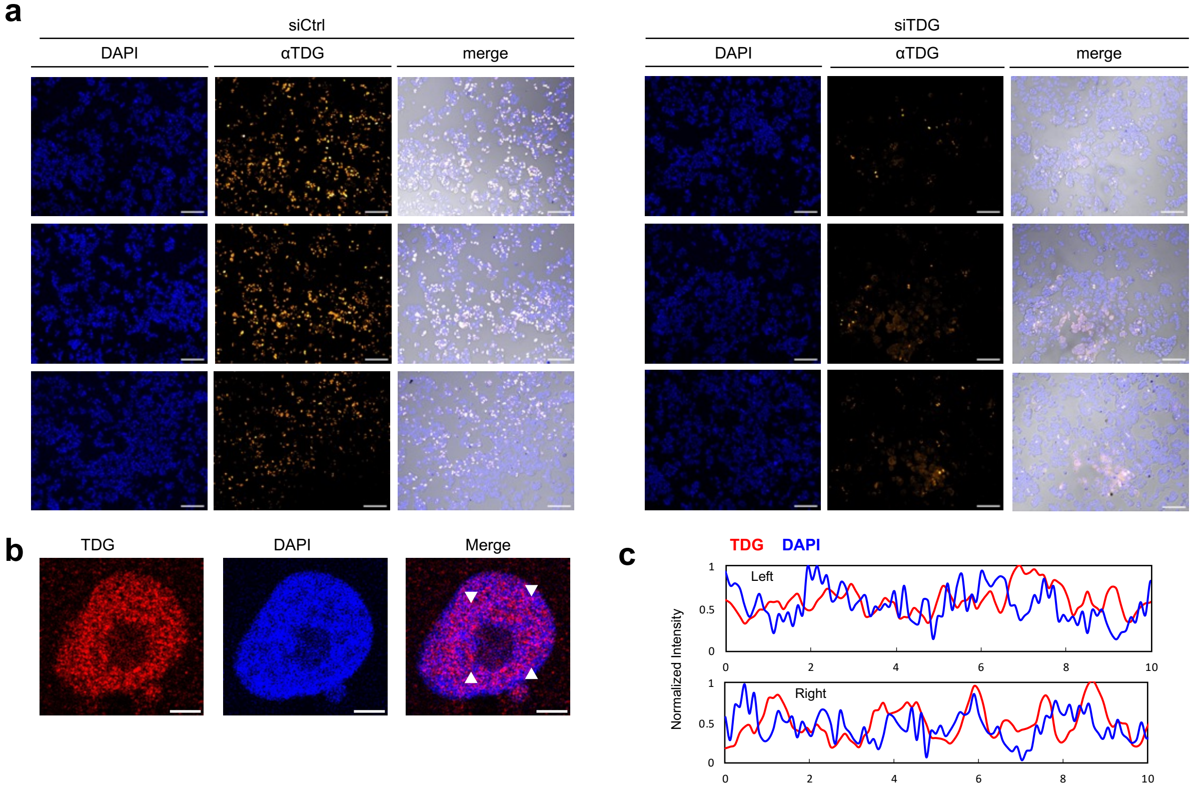

Figure S11. (a) Representative microscopy images of MCF-7 cells immunostained for TDG following treatment with a control (left) or TDG-specific siRNA (right). See Supplementary methods for details. Scale bars: 200 µm (b) Representative fluorescent confocal microscopy images of endogenous TDG (as detected by immunostaining) and DAPI in fixed cells. Scale bars: 5 µm. Pearson's Coefficient: 0.45 ± 0.08 (n = 6 cells) (c) Normalized fluorescence signal intensities of TDG (red) and DAPI (blue) in the nuclei along the line (from left to right) indicated by white arrowheads in panel (b).

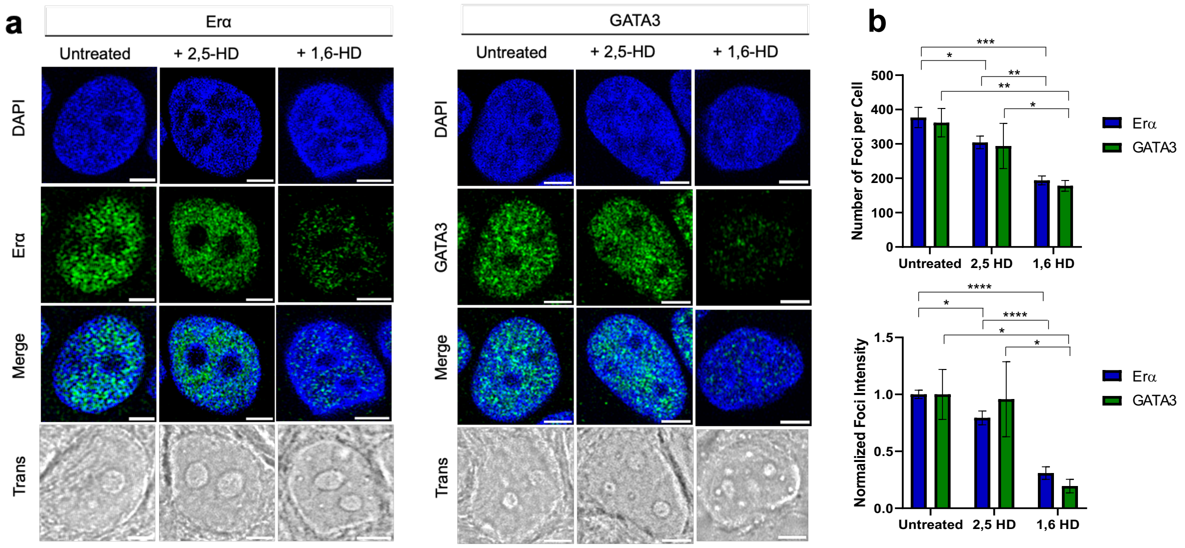

Figure S12. Erα and GATA3 nuclear foci are sensitive to 1,6-HD treatment. (a) Representative fluorescent confocal microscopy images of endogenous Erα (left) and GATA3 (right) showing loss of foci upon 1,6-HD treatment. Erα and GATA3 were visualized by immunostaining fixed cells with their corresponding antibodies. See Supplemental Methods for details. All scale bars: 5 µm. (b) Quantification of foci number and intensity of Erα and GATA3 with either 1,5-HD or 1,6-HD. Foci number are mean ± S.D. (n = 3 cells) for pre- and post-10 min treatment. Erα foci intensity are mean ± S.D. for 377, 305, and 194 individual foci pre-, post-2,5-HD, and post-1,6-HD treatment, respectively. GATA3 foci intensity are mean ± S.D. for 362, 294, and 78 individual foci pre-, post-2,5-HD, and post-1,6-HD treatment, respectively. **P* < 0.05; ***P* < 0.01; ****P* < 0.001 *****P* < 0.0001.

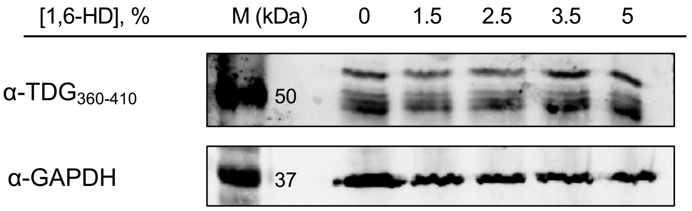

Figure S13. Western blot analysis of endogenous TDG levels (and GADPH control) in MCF-7 cells following treatment with the indicated amount of 1,6-HD after 15 minutes.

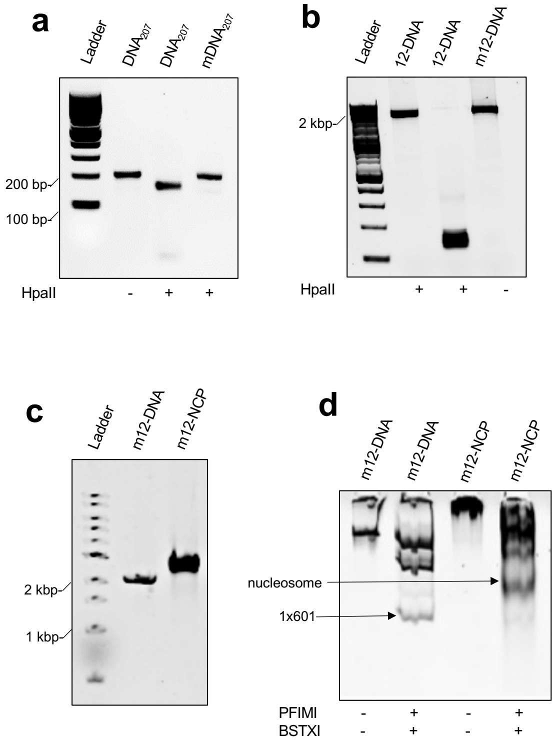

Figure S14. Confirmation of methylated mDNA$_{207}$-Cy5 and 12-NCP-Cy3 following HpaII digestion. (a) Native gel analysis (1% agarose) of DNA$_{207}$ and digestion with the methylation-sensitive restriction enzyme HpaII. (b) Native gel analysis (0.7% Agarose) of 12-NCP-Cy3 and m12-NCP-Cy3 digestion with HpaII. (c) Representative native gel analysis (0.6% agarose) of m12-DNA reconstituted into chromatin with 1.1 molar equivalent of Cy3-labeled histone octamer. (d) Representative restriction digestion analysis (5% native PAGE, 59:1 acrylamide:bisacrylamide) of m12-DNA and m12-NCP-Cy3 demonstrating complete nucleosome occupancy

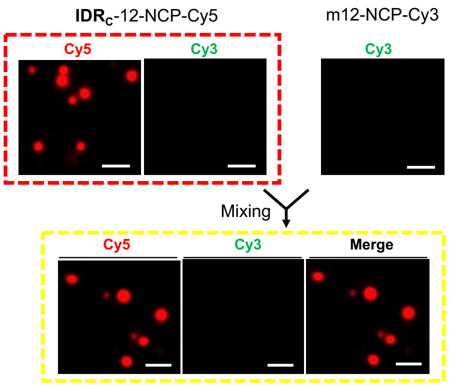

Figure S15. Methylated chromatin m12-NCP-Cy3 (50 nM) is unable to mix with preformed IDR_N-12-NCP-Cy5 droplets generated by mixing 5 µM TDG with 12.5 nM chromatin. Scale bars: 5 µm.

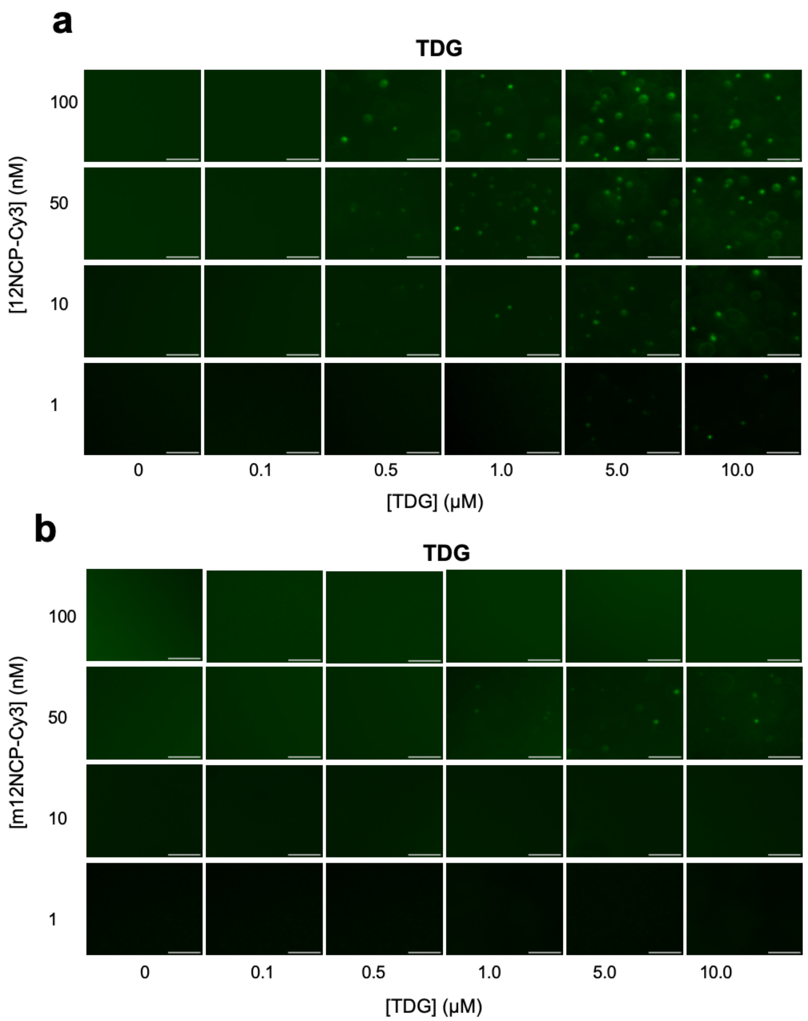

Figure S16. Representative wide-field fluorescent microscopy images used to generate phase diagrams for TDG truncations (Figure 5c). The indicated concentrations of TDG was titrated against a concentration gradient of either 12-NCP-Cy3 (a) or m12-NCP-Cy3 (b) chromatin in 1× LLPS buffer containing 1% PEG and imaged at the glass bottom of a 96-well plate. All scale bar: 25 µm.

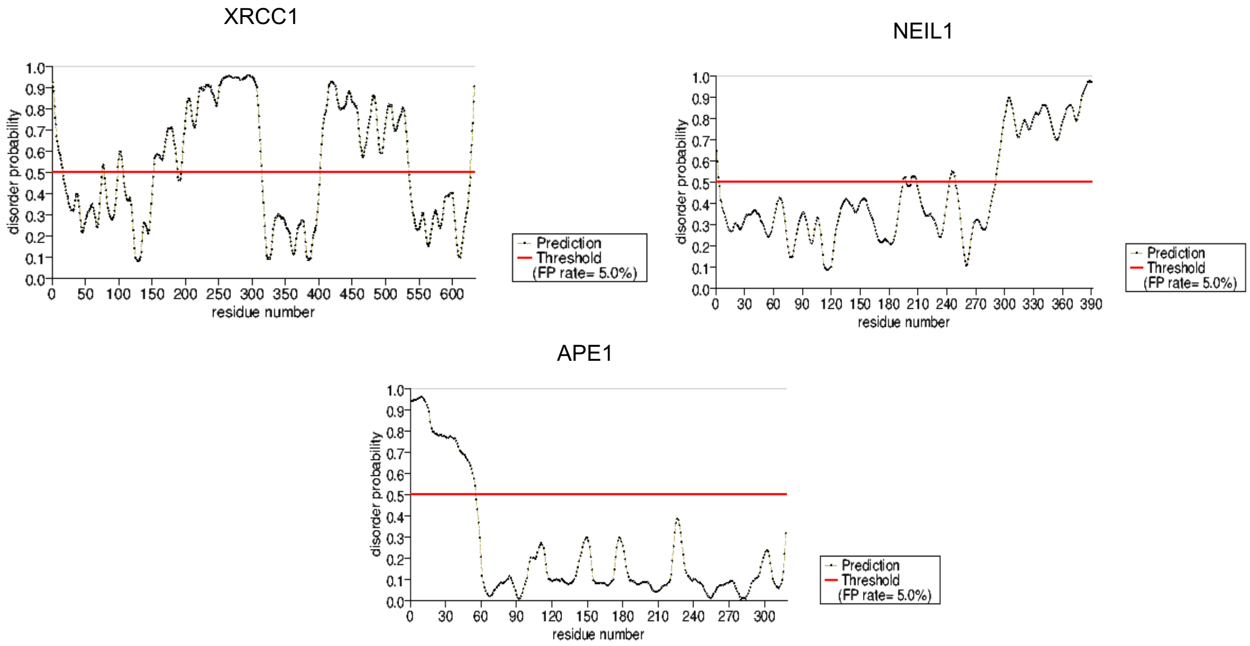

Figure S17. Disorder probability of several base excision repair proteins predicted from PrDOS.[1]

S2. Supplementary Tables

Table S1. Primer sequences used to construct the TFF1 eDNA sequence for chromatin reconstitutions.

Primer Name	Sequence
TFF1eFWD	5'-CTGACTGCTGCCTCTCATCC
TFF1eREV	5'-GGCAGGAAGCTGAGTGTCTT

Table S2. Primer sequences used to construct the 1-601 DNA sequence for *in vitro* LLPS assays.

Primer Name	Sequence
TET2_FWD_PfIMI	5'- AmMC6-GATCCGGAATTCCCAACTAATGGCCGGATCCCCTGGAGAATCCCGGTGCC
BstXI.REV	5'-CGGTATTGCCAAAATCGTGGCGGCAATGGAACAGGATGTATATATCTGACACG

S3. Supplementary References

1. Ishida, T.; Kinoshita, K. (2007) PrDOS: prediction of disordered protein regions from amino acid sequence. *Nucleic Acids Res., 35*, W460-W464.
